# Supplementary material for: Estimates of the basic reproduction number for rubella using seroprevalence data and indicator-based approaches
Source: PLoS Comput Biol. 2022 Mar 3;18(3):e1008858. doi: 10.1371/journal.pcbi.1008858 (PMC8893344; doi:10.1371/journal.pcbi.1008858)
Supplement: S1 Fig — Fig A: Estimates of the basic reproduction number for rubella, as calculated using seroprevalence data (blue circles) and the default approach (red circles) for each study for pessimistic assumptions about the amount of contact between children and adults k = 0.3). The vertical marks indicate R0 as calculated using the regional point estimate for the force of infection. (PDF) [file pcbi.1008858.s001.pdf]

# Estimates of $R_0$ for pessimistic assumptions about contact

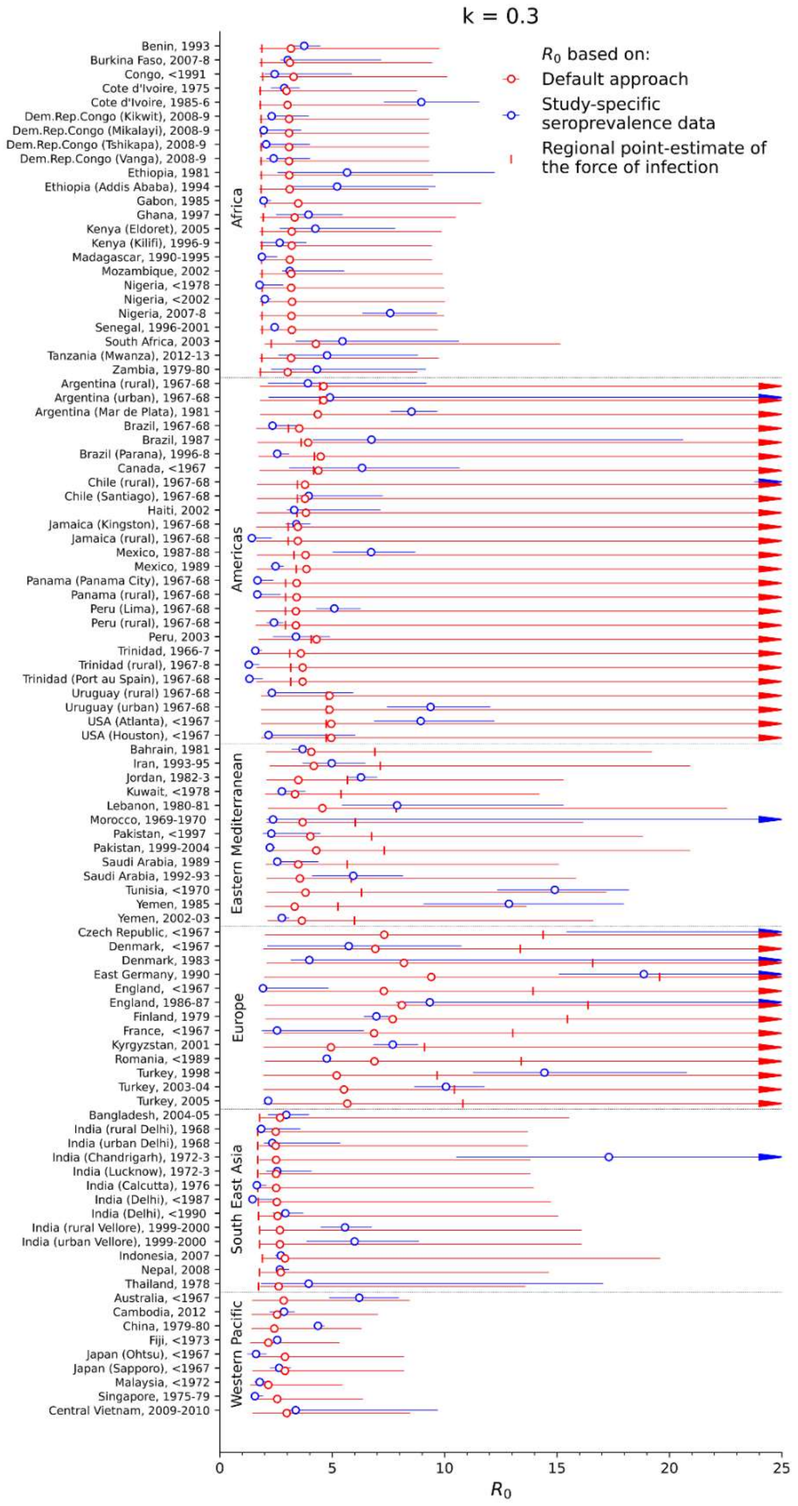


**Fig A:** *Estimates of the basic reproduction number for rubella, as calculated using seroprevalence data (blue circles) and the default approach (red circles) for each study for pessimistic assumptions about the amount of contact between children and adults k=0.3). The vertical marks indicate $R_0$ as calculated using the regional point estimate for the force of infection*
