# Supplementary material for: Estimates of the basic reproduction number for rubella using seroprevalence data and indicator-based approaches
Source: PLoS Comput Biol. 2022 Mar 3;18(3):e1008858. doi: 10.1371/journal.pcbi.1008858 (PMC8893344; doi:10.1371/journal.pcbi.1008858)
Supplement: S2 Table — Table A: Number of studies for which the year in which the indicator was available differed by less than 5 years, 5–10 years or more than 10 years from the year in which the data were collected. For studies with an unspecified year of data collection, the difference was calculated as the difference between the publication year and the year for which the indicator was extracted. The right-most column cites the source from which the data was obtained. Table B: Number of missing indicators for each of the 98 studies. (PDF) [file pcbi.1008858.s003.pdf]

Completeness of the indicator data

Table A: Number of studies for which the year in which the indicator was available differed by less than 5 years, 5-10 years or more than 10 years from the year in which the data were collected. For studies with an unspecified year of data collection, the difference was calculated as the difference between the publication year and the year for which the indicator was extracted. The right-most column cites the source from which the data was obtained.

	**Number of years difference between the year in which the indicator was available and the study year**				**Data source**
	**<5**	**5-10**	**>10**	**Indicator not available**	
**Demographic**					
Proportion of the population aged 0-4	98	0	0	0	[1]
Proportion of the population aged 0-14	98	0	0	0	[1]
Proportion of the population aged 65+	98	0	0	0	[1]
Lifetime risk of maternal death (1 in: rate varies by country)	46	9	43	0	[2]
Probability of dying before age 5 (per 1000 live births)	98	0	0	0	[1]
Crude death rate per 1000 population	98	0	0	0	[1]
Life expectancy at birth (both sexes)	98	0	0	0	[1]
Total fertility rate (live births per woman)	98	0	0	0	[1]
Mean age of child-bearing	98	0	0	0	[1]
Population growth rate (Average annual rate of population change (percentage))	98	0	0	0	[1]
Population density (people per sq. km of land area)	98	0	0	0	[2]
Urban population (% of total)	98	0	0	0	[2]
**Economic**					
Income share held by highest 10%	36	6	50	6	[2]
Income share held by highest 20%	36	6	50	6	[2]
Income share held by lowest 10%	36	6	50	6	[2]
Income share held by lowest 20%	36	6	50	6	[2]
Poverty gap at $1.90 a day (2011 PPP) (%)	40	11	41	6	[2]
Poverty gap at $3.20 a day (2011 PPP) (% of population)	40	11	41	6	[2]

Table A (continued)

	Number of years difference between the year in which the indicator was available and the study year				Data source
	<5	5-10	>10	Indicator not available	
Poverty gap at $5.50 a day (2011 PPP) (% of population)	40	11	41	6	[2]
Poverty gap at national poverty lines (%)	16	7	28	47	[2]
Poverty headcount ratio at $1.90 a day (2011 PPP) (% of population)	39	8	45	6	[2]
Poverty headcount ratio at $3.20 a day (2011 PPP) (% of population)	40	11	41	6	[2]
Poverty headcount ratio at $5.50 a day (2011 PPP) (% of population)	40	11	41	6	[2]
Poverty headcount ratio at national poverty lines (% of population)	27	8	40	23	[2]
GDP per capita, PPP (constant 2011 international $)	46	9	43	0	[2]
GDP per capita, PPP (current international $)	46	9	43	0	[2]
HDI	41	12	45	0	[3]
GDP per capita, current prices (Purchasing power parity; international dollars per capita)	61	6	31	0	[4]
GDP per capita (1990 Int. GK$)	97	0	0	1	[5]
**Educational**					
Adjusted net enrollment rate, primary (% of primary school age children)	25	12	56	5	[2]
Educational attainment, at least completed upper secondary, population 25+, female (%) (cumulative)	27	15	46	10	[2]
Educational attainment, at least completed upper secondary, population 25+, male (%) (cumulative)	27	15	46	10	[2]
Educational attainment, at least completed upper secondary, population 25+, total (%) (cumulative)	29	15	44	10	[2]
**Employment**					[2]
Unemployment, total (% of total labor force) (modeled ILO estimate)	45	9	44	0	[2]
Unemployment, total (% of total labor force) (national estimate)	63	18	17	0	[2]
Employment to population ratio, 15+, female (%) (modeled ILO estimate)	45	9	44	0	[2]

Table A (continued)

	Number of years difference between the year in which the indicator was available and the study year				Data source
	<5	5-10	>10	Indicator not available	
Employment to population ratio, 15+, female (%) (national estimate)	32	16	37	13	[2]
Employment to population ratio, ages 15-24, female (%) (modeled ILO estimate)	45	9	44	0	[2]
Employment to population ratio, ages 15-24, female (%) (national estimate)	18	10	52	18	[2]
**Health-related**					
Low-birthweight babies (% of births)	32	11	55	0	[2]
Prevalence of undernourishment (% of population)	39	6	32	21	[2]
Prevalence of underweight, weight for age (% of children under 5)	48	20	26	4	[2]
Health expenditure, total (% of GDP)	36	13	49	0	[2]
Immunization, DPT (% of children ages 12-23 months)	62	6	30	0	[2]
Immunization, HepB3 (% of one-year-old children)	28	10	53	7	[2]
Immunization, measles (% of children ages 12-23 months)	60	5	33	0	[2]
Exclusive breastfeeding (% of children under 6 months)	31	10	42	15	[2]
Physicians (per 1,000 people)	95	1	2	0	[2]
Number of doctors' consultations	13	3	9	73	[6]
HiB vaccination coverage	17	13	66	2	[7]
**Housing**					
Average number of people per room in occupied housing unit	5	1	6	86	[8]
Population living in slums (% of urban population)	39	3	33	23	[2]
Number of households All households - Per capita	12	4	45	37	[9]
Number of households 1 person - Per capita	9	2	45	42	[9]
Number of households 1 person - Proportion over All households	9	2	45	42	[9]
Number of households 2 persons - Per capita	9	2	45	42	[9]
Number of households 2 persons - Proportion over All households	9	2	45	42	[9]

Table A (continued)

	Number of years difference between the year in which the indicator was available and the study year				Data source
	<5	5-10	>10	Indicator not available	
Number of households 3 persons - Per capita	9	2	45	42	[9]
Number of households 3 persons - Proportion over All households	9	2	45	42	[9]
Number of households 4 persons - Per capita	9	2	45	42	[9]
Number of households 4 persons - Proportion over All households	9	2	45	42	[9]
Number of households 5 persons - Per capita	9	2	45	42	[9]
Number of households 5 persons - Proportion over All households	9	2	45	42	[9]
Number of households 6 persons and over - Per capita	9	2	42	45	[9]
Number of households 6 persons and over - Proportion over All households	9	2	42	45	[9]
Total population in a household, both sexes	2	2	22	72	[10]

Table B: Number of missing indicators for each of the 98 studies

Study Name	Number of missing indicator values
Benin, 1993[11]	15
Burkina Faso, 2007-8[12]	2
Congo, <1991[13]	18
Cote d'Ivoire, 1975[14]	18
Cote d'Ivoire, 1985-6[15]	18
Democratic Republic of the Congo (Kikwit), 2008-9[16]	20
Democratic Republic of the Congo (Mikalayi), 2008-9[16]	20
Democratic Republic of the Congo (Tshikapa), 2008-9[16]	20
Democratic Republic of the Congo (Vanga), 2008-9[16]	20
Ethiopia, 1981[17]	3
Ethiopia (Addis Ababa), 1994[18]	3
Gabon, 1985[19]	20
Ghana, 1997[20]	2
Kenya (Eldoret), 2005[21]	18
Kenya (Kilifi), 1996-9[22, 23]	18
Madagascar, 1990-1995[24]	19
Mozambique, 2002[25]	16
Nigeria, <1978[26]	21
Nigeria, <2002[27]	21
Nigeria, 2007-8[28]	21
Senegal, 1996-2001[29]	16
South Africa, 2003[30]	1
Tanzania (Mwanza), 2012-13[31]	16
Zambia, 1979-80[32]	5
Argentina (rural), 1967-68[33]	4
Argentina (urban), 1967-68[33]	4
Argentina (Mar de Plata), 1981[34]	4
Brazil, 1967-68[33]	2
Brazil, 1987[35]	2
Brazil (Parana), 1996-8[36]	2
Canada, <1967[37]	6
Chile (rural), 1967-68[33]	3
Chile (Santiago), 1967-68[33]	3
Haiti, 2002[38]	17
Jamaica (Kingston), 1967-68[33]	6
Jamaica (rural), 1967-68[33]	6
Mexico, 1987-88[39]	1
Mexico, 1989[40]	1
Panama (Panama City), 1967-68[33]	17

Panama (rural), 1967-68[33]	17
Peru (Lima), 1967-68[33]	3
Peru (rural), 1967-68[33]	3
Peru, 2003[41]	3
Trinidad, 1966-7[42]	7
Trinidad (rural), 1967-8[33]	7
Trinidad (Port au Spain), 1967-68[33]	7
Uruguay (rural) 1967-68[33]	3
Uruguay (urban) 1967-68[33]	3
USA (Atlanta), <1967[37]	6
USA (Houston), <1967[37]	6
Bahrain, 1981[43]	30
Iran, 1993-95[44]	5
Jordan, 1982-3[45]	16
Kuwait, <1978[46]	30
Lebanon, 1980-81[47]	16
Morocco, 1969-1970[48]	19
Pakistan, <1997[49]	16
Pakistan, 1999-2004[50]	16
Saudi Arabia, 1989[51]	28
Saudi Arabia, 1992-93[52]	28
Tunisia, <1970[53]	17
Yemen, 1985[54]	19
Yemen, 2002-03[55]	19
Czech Republic, <1967[37]	5
Denmark, <1967[37]	21
Denmark, 1983[56]	21
East Germany, 1990[57]	5
England, <1967[37]	6
England, 1986-87[58]	6
Finland, 1979[59]	7
France, <1967[37]	20
Kyrgyzstan, 2001[60]	3
Romania, <1989[61]	4
Turkey, 1998[62]	14
Turkey, 2003-04[63]	14
Turkey, 2005[64]	14
Bangladesh, 2004-05[65]	4
India (rural Delhi), 1968[66]	3
India (urban Delhi), 1968[66]	3
India (Chandrigarh), 1972-3[66]	3
India (Lucknow), 1972-3[66]	3
India (Calcutta), 1976[67]	3
India (Delhi), <1987[68]	3

India (Delhi), <1990[69]	3
India (rural Vellore), 1999-2000[70]	3
India (urban Vellore), 1999-2000[70]	3
Indonesia, 2007 (*S Reef, personal communication, March 2015*)	16
Nepal, 2008[71]	4
Thailand, 1978[72]	5
Australia, <1967[37]	6
Cambodia, 2012[73]	25
China, 1979-80[74]	9
Fiji, <1973[75]	19
Japan (Sapporo), <1967[37]	7
Japan (Ohtsu), <1967[37]	7
Malaysia, <1972[76]	3
Singapore, 1975-79[77]	19
Central Vietnam, 2009-2010[78]	3

References

1. United Nations, Department of Economic and Social Affairs, Population Division, World Population Prospects. https://population.un.org/wpp/Download/Archive/Standard/2017.
2. The World Bank Data Catalog, World Development Indicators. https://datacatalog.worldbank.org/dataset/world-development-indicators/resource/08feba-8a62-11e6-ae22-56b6b64900022017.
3. UNITED NATIONS DEVELOPMENT PROGRAMME Human Development Reports Table 2. Human Development Index Trends, 1990-2015 http://hdr.undp.org/en/composite/trends2016.
4. IMF DataMapper. https://www.imf.org/external/datamapper/PPPPC@WEO/OEMDC/ADVEC/WEOWORLD/.
5. University of Groningen Growth and Development Centre, Maddison Database 2010. https://www.rug.nl/ggdc/historicaldevelopment/maddison/data/md2010_vertical.xlsx.
6. Organisation for Economic Co-operation and Development, OECD.Stat, Health Care Utilisation: Consultations. https://stats.oecd.org/index.aspx?queryid=30161.
7. WHO Global Health Observatory data repository Hib (Hib3) Immunization coverage estimates by country. https://apps.who.int/gho/data/node.main.A829?lang=en.
8. WHO European Health Information Gateway Health for All explorer. https://gateway.euro.who.int/en/hfa-explorer/#CtIn99Lev6.
9. United Nations, Department of Economic and Social Affairs, Statistics Division. Demographic Statistics Database, Households by age and sex of reference person and by size of household. http://data.un.org/Data.aspx?d=POP&f=tableCode:50.
10. United Nations, Department of Economic and Social Affairs, Statistics Division, Demographic Statistics Database, Population in households by type of household, age and sex. http://data.un.org/Data.aspx?d=POP&f=tableCode:329.
11. Rodier MH, Berthonneau J, Bourgoin A, Giraudeau G, Agius G, Burucoa C, et al. Seroprevalences of Toxoplasma, malaria, rubella, cytomegalovirus, HIV and treponemal infections among pregnant women in Cotonou, Republic of Benin. Acta tropica. 1995;59(4):271-7. Epub 1995/08/01. PubMed PMID: 8533662.
12. Tahita MC, Hubschen JM, Tarnagda Z, Ernest D, Charpentier E, Kremer JR, et al. Rubella seroprevalence among pregnant women in Burkina Faso. BMC Infect Dis. 2013;13:164. doi: 10.1186/1471-2334-13-164. PubMed PMID: 23556510; PubMed Central PMCID: PMCPMC3623657.
13. Yala F, Biendo M, Odongo I, Kounkou R. [Virological and bacteriological study of materno-fetal infections in Brazzaville]. Bulletin de la Societe de pathologie exotique (1990). 1991;84(5 Pt 5):627-34. Epub 1991/01/01. PubMed PMID: 1819414.
14. Vrinat M, Dutertre J, Helies H, Ropero P. [A serological survey of rubella among pregnant women in Abidjan (author's transl)]. Medecine tropicale : revue du Corps de sante colonial. 1978;38(1):53-7. Epub 1978/01/01. PubMed PMID: 214661.
15. Ouattara SA, Brettes JP, Kodjo R, Penali K, Gershy-Damet G, Sangare A, et al. [Seroepidemiology of rubella in the Ivory Coast. Geographic distribution]. Bulletin de la Societe de pathologie exotique et de ses filiales. 1987;80(4):655-64. Epub 1987/01/01. PubMed PMID: 2830995.
16. Alleman MM, Wannemuehler KA, Hao L, Perelygina L, Icenogle JP, Vynnycky E, et al. Estimating the burden of rubella virus infection and congenital rubella syndrome through a

rubella immunity assessment among pregnant women in the Democratic Republic of the Congo: Potential impact on vaccination policy. Vaccine. 2016;34(51):6502-11. doi: 10.1016/j.vaccine.2016.10.059. PubMed PMID: 27866768.
17. Sandow D, Okubagzhi GS, Arnold U, Denkmann N. Seroepidemiological study in rubella in pregnant women in Gondar Region, northern Ethiopia. Ethiopian medical journal. 1982;20(4):173-8. Epub 1982/10/01. PubMed PMID: 7140729.
18. Cutts FT, Abebe A, Messele T, Dejene A, Enquselassie F, Nigatu W, et al. Sero-epidemiology of rubella in the urban population of Addis Ababa, Ethiopia. Epidemiol Infect. 2000;124(3):467-79. Epub 2000/09/12. PubMed PMID: 10982071; PubMed Central PMCID: PMC2810933.
19. Mefane C. Rubella antibodies in 1737 girls and women in Gabon. Afrique Medicale. 1985;24(226):29-32. PubMed PMID: 19852020650.
20. Lawn JE, Reef S, Baffoe-Bonnie B, Adadevoh S, Caul EO, Griffin GE. Unseen blindness, unheard deafness, and unrecorded death and disability: congenital rubella in Kumasi, Ghana. American journal of public health. 2000;90(10):1555-61. Epub 2000/10/13. PubMed PMID: 11029988; PubMed Central PMCID: PMCPMC1446363.
21. Kombich JM, PC; Borus, PK Seroprevalence of Natural Rubella Antibodies among Antenatal Attendees at Moi Teaching and Referral Hospital, Eldoret, Kenya. Journal of Immunological Techniques in Infectious Diseases. 2012;1(1).
22. Cumberland P, Shulman CE, Maple PA, Bulmer JN, Dorman EK, Kawuondo K, et al. Maternal HIV infection and placental malaria reduce transplacental antibody transfer and tetanus antibody levels in newborns in Kenya. J Infect Dis. 2007;196(4):550-7. Epub 2007/07/13. doi: JID37995 [pii] 10.1086/519845. PubMed PMID: 17624840.
23. Scott S, Cumberland P, Shulman CE, Cousens S, Cohen BJ, Brown DW, et al. Neonatal measles immunity in rural Kenya: the influence of HIV and placental malaria infections on placental transfer of antibodies and levels of antibody in maternal and cord serum samples. J Infect Dis. 2005;191(11):1854-60. Epub 2005/05/05. doi: JID33850 [pii] 10.1086/429963. PubMed PMID: 15871118.
24. Dromigny JA, Pecarrere JL, Ollivier G, Leroy F, Zeller HG. [Seroprevalence of rubella in pregnant women at Antananarivo. Study of 853 sera at the Pasteur Institute in Madagascar]. Archives de l'Institut Pasteur de Madagascar. 1996;63(1-2):53-5. Epub 1996/01/01. PubMed PMID: 12463018.
25. Barreto J, Sacramento I, Robertson SE, Langa J, de Gourville E, Wolfson L, et al. Antenatal rubella serosurvey in Maputo, Mozambique. Trop Med Int Health. 2006;11(4):559-64. Epub 2006/03/24. doi: 10.1111/j.1365-3156.2006.01577.x. PubMed PMID: 16553940.
26. Odelola HA. Rubella haemagglutination inhibiting antibodies in females of child-bearing age in western Nigeria. Journal of hygiene, epidemiology, microbiology, and immunology. 1978;22(2):190-4. Epub 1978/01/01. PubMed PMID: 570582.
27. Bukbuk DN, el Nafaty AU, Obed JY. Prevalence of rubella-specific IgG antibody in non-immunized pregnant women in Maiduguri, north eastern Nigeria. Central European journal of public health. 2002;10(1-2):21-3. Epub 2002/07/05. PubMed PMID: 12096678.
28. Amina MD, Oladapo S, Habib S, Adebola O, Bimbo K, Daniel A. Prevalence of rubella IgG antibodies among pregnant women in Zaria, Nigeria. International Health. 2010;2(2):156-9. PubMed PMID: 20103221973.
29. Dromigny JA, Nabeth P, Perrier Gros Claude JD. Evaluation of the seroprevalence of rubella in the region of Dakar (Senegal). Trop Med Int Health. 2003;8(8):740-3. Epub 2003/07/19. PubMed PMID: 12869096.
30. Corcoran C, Hardie DR. Seroprevalence of rubella antibodies among antenatal patients in the Western Cape. South African medical journal = Suid-Afrikaanse tydskrif vir geneeskunde. 2005;95(9):688-90. Epub 2005/12/06. PubMed PMID: 16327929.

31. Mwambe B, Mirambo MM, Mshana SE, Massinde AN, Kidenya BR, Michael D, et al. Sero-positivity rate of rubella and associated factors among pregnant women attending antenatal care in Mwanza, Tanzania. BMC Pregnancy Childbirth. 2014;14:95. doi: 10.1186/1471-2393-14-95. PubMed PMID: 24589180; PubMed Central PMCID: PMCPMC3975942.
32. Watts T. Rubella antibodies in a sample of Lusaka mothers. Medical journal of Zambia. 1983;17(4):109-10. Epub 1983/10/01. PubMed PMID: 6680556.
33. Dowdle WR, Ferrera W, De Salles Gomes LF, King D, Kourany M, Madalengoitia J, et al. WHO collaborative study on the sero-epidemiology of rubella in Caribbean and Middle and South American populations in 1968. Bull World Health Organ. 1970;42(3):419-22. Epub 1970/01/01. PubMed PMID: 5310208; PubMed Central PMCID: PMCPMC2427532.
34. Pereira F, Uez O. Rubella antibodies in female applicants for premarital health certificates in Mar del Plata, Argentina. Bull Pan Am Health Organ. 1986;20(2):179-85. Epub 1986/01/01. PubMed PMID: 3768598.
35. Souza VA, Moraes JC, Sumita LM, Camargo MC, Fink MC, Hidalgo NT, et al. Prevalence of rubella antibodies in a non-immunized urban population, Sao Paulo, Brazil. The Division of Immunization, CVE. Revista do Instituto de Medicina Tropical de Sao Paulo. 1994;36(4):373-6. Epub 1994/07/01. PubMed PMID: 7732269.
36. Reiche EM, Morimoto HK, Farias GN, Hisatsugu KR, Geller L, Gomes AC, et al. [Prevalence of American trypanosomiasis, syphilis, toxoplasmosis, rubella, hepatitis B, hepatitis C, human immunodeficiency virus infection, assayed through serological tests among pregnant patients, from 1996 to 1998, at the Regional University Hospital Norte do Parana]. Rev Soc Bras Med Trop. 2000;33(6):519-27. Epub 2001/02/15. doi: S0037-86822000000600002 [pii]. PubMed PMID: 11175581.
37. Rawls WE, Melnick JL, Bradstreet CM, Bailey M, Ferris AA, Lehmann NI, et al. WHO collaborative study on the sero-epidemiology of rubella. Bull World Health Organ. 1967;37(1):79-88. Epub 1967/01/01. PubMed PMID: 5300057; PubMed Central PMCID: PMC2554213.
38. Desinor OY, Anselme RJ, Laender F, Saint-Louis C, Bien-Aime JE. Seroprevalence of antibodies against rubella virus in pregnant women in Haiti. Rev Panam Salud Publica. 2004;15(3):147-50. Epub 2004/04/21. PubMed PMID: 15096284.
39. Gutierrez Trujillo G, Munoz O, Tapia Conyer R, Bustamante Calvillo ME, Alvarez y Munoz MT, Guiscafre Gallardo JP, et al. [The seroepidemiology of rubella in Mexican women. A national probability survey]. Salud publica de Mexico. 1990;32(6):623-31. Epub 1990/11/01. PubMed PMID: 2089638.
40. Yamamoto L, Mejia E, Lopez RM, Gallardo E, Gomez B. Susceptibility to rubella infection in females at high risk. Immune protection associated to population density. Tropical and geographical medicine. 1995;47(6):235-8. Epub 1995/01/01. PubMed PMID: 8650731.
41. Suarez-Ognio L, Adrianzen A, Ortiz A, Martinez C, Whittembury A, Cabezudo E, et al. A rubella serosurvey in postpartum women in the three regions of Peru. Rev Panam Salud Publica. 2007;22(2):110-7. Epub 2007/11/03. PubMed PMID: 17976277.
42. Pitts OM, Ravenel JM, Finklea JF. Rubella immunity in Trinidad. American journal of epidemiology. 1969;89(3):271-6. Epub 1969/03/01. PubMed PMID: 5773423.
43. Dutta SR, Atrash HK, Mathew L, Mathew PP, Mahmood RA. Seroepidemiology of rubella in Bahrain. Int J Epidemiol. 1985;14(4):618-23. Epub 1985/12/01. PubMed PMID: 4086149.
44. Modarres S, Modarres S, Oskoii NN. The immunity of children and adult females to rubella virus infection in Tehran. Iranian journal of medical sciences. 1996;21:69-73.
45. El-Khateeb MS, Tarawneh MS, Hijazi S, Kahwaji L. Seroimmunity to rubella virus in Jordanians. Public Health. 1983;97(4):204-7. Epub 1983/07/01. PubMed PMID: 6622640.

46. Hathout H, Al-Nakib W, Lilley H, Abo-Ahmed HS, Nosseir AF. Seroepidemiology of rubella in Kuwait: an alternative vaccination policy. Int J Epidemiol. 1978;7(1):49-53. Epub 1978/03/01. PubMed PMID: 659049.
47. Bedrossian NK, Matossian R. Is there a rubella problem in Lebanon? Lebanese Medical Journal. 1985;35(1):31-8.
48. Nejmi S. [Immunologic survey of rubella in Moroccan women in the Rabat region (study of antibodies inhibiting hemagglutination in 548 serums)]. Maroc medical. 1972;52(559):420-5. Epub 1972/07/01. PubMed PMID: 4642407.
49. Iqbal A, Bokhari S. Occurrence of rubella antibody IgG in the general population. Mother and Child. 1997;35(1):17-22.
50. Ahmed R, Hashmi K, Ullah SE, Khanum T, Rafia A. Study of Prevalence of Immune Status in Adult Females For Rubella Virus Infection. Pakistan Journal of Biological Sciences. 2006;9(5):816.
51. Hossain A. Seroepidemiology of rubella in Saudi Arabia. Journal of tropical pediatrics. 1989;35(4):169-70. Epub 1989/08/01. PubMed PMID: 2810460.
52. Saeed A, Abu-Shagra S, Al-Rasheed R. Congenital Rubella Syndrome - revisited (letter). Saudi Med J. 1993;14(25-26).
53. Nabli B. [Seroepidemiology of rubella in Tunisia]. Bull World Health Organ. 1970;42(6):891-6. Epub 1970/01/01. PubMed PMID: 5312251; PubMed Central PMCID: PMCPMC2427574.
54. Strauss J, Dobahi SS, Danes L, Kopecky K, Svandova E. Serological survey of rubella in Yemen in 1985. Journal of hygiene, epidemiology, microbiology, and immunology. 1989;33(2):163-7. Epub 1989/01/01. PubMed PMID: 2768819.
55. Sallam TA, Al-Jaufy AY, Al-Shaibany KS, Ghauth AB, Best JM. Prevalence of antibodies to measles and rubella in Sana'a, Yemen. Vaccine. 2006;24(37-39):6304-8. Epub 2006/07/04. doi: 10.1016/j.vaccine.2006.05.083. PubMed PMID: 16815602.
56. Glikmann G, Petersen I, Mordhorst CH. Prevalence of IgG-antibodies to mumps and measles virus in non-vaccinated children. Dan Med Bull. 1988;35(2):185-7. Epub 1988/04/01. PubMed PMID: 3359817.
57. Edmunds WJ, Gay NJ, Kretzschmar M, Pebody RG, Wachmann H. The pre-vaccination epidemiology of measles, mumps and rubella in Europe: implications for modelling studies. Epidemiol Infect. 2000;125(3):635-50. Epub 2001/02/24. PubMed PMID: 11218214; PubMed Central PMCID: PMCPMC2869647.
58. Morgan-Capner P, Wright J, Miller CL, Miller E. Surveillance of antibody to measles, mumps, and rubella by age. BMJ. 1988;297(6651):770-2. Epub 1988/09/24. PubMed PMID: 3142541; PubMed Central PMCID: PMC1834398.
59. Ukkonen P. Rubella immunity and morbidity: impact of different vaccination programs in Finland 1979-1992. Scand J Infect Dis. 1996;28(1):31-5. Epub 1996/01/01. PubMed PMID: 9122630.
60. Malakmadze N, Zimmerman LA, Uzicanin A, Shteinke L, Caceres VM, Kasymbekova K, et al. Development of a rubella vaccination strategy: contribution of a rubella susceptibility study of women of childbearing age in Kyrgyzstan, 2001. Clinical infectious diseases : an official publication of the Infectious Diseases Society of America. 2004;38(12):1780-3. Epub 2004/07/01. doi: 10.1086/421018. PubMed PMID: 15227627.
61. Dumitrescu R, Mateescu M, Gaicu N, Comanescu D. Evaluation of the anti-rubella immunity levels on a lot of 5,000 sera from women at procreative age, tested by HAI, in Romania. Archives roumaines de pathologie experimentales et de microbiologie. 1989;48(3):253-63. Epub 1989/07/01. PubMed PMID: 2519635.
62. Aksakal FN, Maral I, Cirak MY, Aygun R. Rubella seroprevalence among women of childbearing age residing in a rural region: is there a need for rubella vaccination in Turkey? Jpn J Infect Dis. 2007;60(4):157-60. Epub 2007/07/24. PubMed PMID: 17642522.

63. Pehlivan E, Karaoglu L, Ozen M, Gunes G, Tekerekoglu MS, Genc MF, et al. Rubella seroprevalence in an unvaccinated pregnant population in Malatya, Turkey. Public Health. 2007;121(6):462-8. Epub 2007/01/16. doi: S0033-3506(06)00327-1 [pii] 10.1016/j.puhe.2006.09.021. PubMed PMID: 17222875.
64. Sasmaz T, Kurt AO, Ozturk C, Bugdayci R, Oner S. Rubella seroprevalence in women in the reproductive period, Mersin, Turkey. Vaccine. 2007;25(5):912-7. Epub 2006/10/20. doi: S0264-410X(06)01010-3 [pii] 10.1016/j.vaccine.2006.09.033. PubMed PMID: 17049680.
65. Nessa A, Islam MN, Tabassum S, Munshi SU, Ahmed M, Karim R. Seroprevalence of rubella among urban and rural Bangladeshi women emphasises the need for rubella vaccination of pre-pubertal girls. Indian journal of medical microbiology. 2008;26(1):94-5. Epub 2008/01/30. PubMed PMID: 18227617.
66. Seth P, Manjunath N, Balaya S. Rubella infection: the Indian scene. Rev Infect Dis. 1985;7 Suppl 1:S64-7. Epub 1985/03/01. PubMed PMID: 4001736.
67. Chakravarty MS, Gupta B, Das BC, Mukherjee MK, Mitra AC, Sarkar JK. Seroepidemiological study of rubella in Calcutta. The Indian journal of medical research. 1976;64(1):87-92. Epub 1976/01/01. PubMed PMID: 1270103.
68. Khare S, Banerjee K, Padubidri V, Rai A, Kumari S, Kumari S. Lowered immunity status of rubella virus infection in pregnant women. The Journal of communicable diseases. 1987;19(4):391-5. Epub 1987/12/01. PubMed PMID: 3507446.
69. Khare S, Gupta HL, Banerjee K, Kumari S, Kumari S, Gupta HL. Seroimmunity to rubella virus infection in young adult females in Delhi. The Journal of communicable diseases. 1990;22(4):279-80. Epub 1990/12/01. PubMed PMID: 2098435.
70. Brown DWJ, Cutts FT, Joseph A. An evaluation of complementary epidemiological methods in a defined population in Southern India for estimating the burden of Congenital Rubella Syndrome. 2004.
71. Upreti SR, Thapa K, Pradhan YV, Shakya G, Sapkota YD, Anand A, et al. Developing rubella vaccination policy in Nepal--results from rubella surveillance and seroprevalence and congenital rubella syndrome studies. J Infect Dis. 2011;204 Suppl 1:S433-8. Epub 2011/06/17. doi: 10.1093/infdis/jir078. PubMed PMID: 21666196.
72. Desudchit P, Chatiyanonda K, Bhamornsathit S. Rubella antibody among Thai women of childbearing age. Southeast Asian J Trop Med Public Health. 1978;9(3):312-6. Epub 1978/09/01. PubMed PMID: 311950.
73. Mao B, Chheng K, Wannemuehler K, Vynnycky E, Buth S, Soeung SC, et al. Immunity to polio, measles and rubella in women of child-bearing age and estimated congenital rubella syndrome incidence, Cambodia, 2012. Epidemiol Infect. 2014:1-10. Epub 2014/11/07. doi: S0950268814002817 [pii] 10.1017/S0950268814002817. PubMed PMID: 25373419.
74. Wannian S. Rubella in the People's Republic of China. Reviews of Infectious Diseases. 1985;7(Supp 1):S72.
75. Macnamara FN, Mitchell R, Miles JA. A study of immunity to rubella in villages in the Fiji islands using the haemagglutination inhibition test. J Hyg (Lond). 1973;71(4):825-31. Epub 1973/12/01. PubMed PMID: 4520516; PubMed Central PMCID: PMC2130425.
76. Lam SK. The seroepidemiology of rubella in Kuala Lumpur, West Malaysia. Bull World Health Organ. 1972;47(1):127-9. Epub 1972/01/01. PubMed PMID: 4538899; PubMed Central PMCID: PMCPMC2480804.
77. Doraisingham S, Goh KT. The rubella immunity of women of child-bearing age in Singapore. Annals of the Academy of Medicine, Singapore. 1981;10(2):238-41. Epub 1981/04/01. PubMed PMID: 7332291.
78. Miyakawa M, Yoshino H, Yoshida LM, Vynnycky E, Motomura H, Tho le H, et al. Seroprevalence of rubella in the cord blood of pregnant women and congenital rubella

incidence in Nha Trang, Vietnam. Vaccine. 2014;32(10):1192-8. Epub 2013/09/12. doi: S0264-410X(13)01188-2 [pii] 10.1016/j.vaccine.2013.08.076. PubMed PMID: 24021315.
