# Supplementary material for: Estimates of the basic reproduction number for rubella using seroprevalence data and indicator-based approaches
Source: PLoS Comput Biol. 2022 Mar 3;18(3):e1008858. doi: 10.1371/journal.pcbi.1008858 (PMC8893344; doi:10.1371/journal.pcbi.1008858)
Supplement: S3 Table — Table A: Estimates of the mean square error associated with R0 estimates. Column 2 shows the mean square error of R0, as calculated using the default approach against R0 calculated using study-specific seroprevalence data. Columns 2–7 hold the minimum, median, maximum 2.5th and 95.5th percentiles of the MSE values between the 1000 bootstrap-derived values of R0 calculated using the regional collection of force of infection bootstrap values and the 1000 bootstrap-derived study-specific R0 values. The final column holds the MSE between study-specific R0 and the regional point estimate of R0. (PDF) [file pcbi.1008858.s004.pdf]

Estimates of the mean square error associated with R_0 estimates

Table A: Estimates of the mean square error associated with R_0 estimates. Column 2 shows the mean square error of R_0, as calculated using the default approach against R_0 calculated using study-specific seroprevalence data. Columns 2-7 hold the minimum, median, maximum 2.5th and 95.5th percentiles of the MSE values between the 1000 bootstrap-derived values of R_0 calculated using the regional collection of force of infection bootstrap values and the 1000 bootstrap-derived study-specific R_0 values. The final column holds the MSE between study-specific R_0 and the regional point estimate of R_0.

	**R_0 from study-specific seroprevalence data vs default R_0 estimate**	**Min**	**2.5 %**	**Median**	**97.5%**	**Max**	**R_0 from study-specific seroprevalence data vs R_0 from regional point estimate of the force of infection**
**All countries**	6.97	4.76	7.31	16.03	125.75	1513.39	8.31
**Africa**	1.03	0.73	1.01	2.16	16.23	65.14	2.23
**Americas**	8.72	4.62	6.60	12.04	298.38	1992.15	9.36
**Eastern Mediterranean**	5.01	2.73	3.88	6.46	75.69	3183.24	4.61
**Europe**	22.56	6.83	13.16	44.09	694.90	11359.21	28.10
**South East Asia**	4.71	1.33	2.38	7.65	133.70	440.50	6.14
**Western Pacific**	1.36	0.86	1.04	2.44	18.57	281.34	1.39
