# Supplementary material for: Estimates of the basic reproduction number for rubella using seroprevalence data and indicator-based approaches
Source: PLoS Comput Biol. 2022 Mar 3;18(3):e1008858. doi: 10.1371/journal.pcbi.1008858 (PMC8893344; doi:10.1371/journal.pcbi.1008858)
Supplement: S4 Table — Table A: Summary of the correlation coefficients and MIC for the association between the basic reproduction number and the indicators. ranked in decreasing order. The columns labelled “CC” hold the coefficient, with the 95% range obtained by bootstrapping; the column labelled “N” holds the number of data points used to calculate the coefficient. (PDF) [file pcbi.1008858.s005.pdf]

Correlation between R_0 and the indicators

Table A: Summary of the correlation coefficients and MIC for the association between the basic reproduction number and the indicators. ranked in decreasing order. The columns labelled "CC" hold the coefficient, with the 95% range obtained by bootstrapping; the column labelled "N" holds the number of data points used to calculate the coefficient.

Rank	Pearson			Spearman			MIC		
	**Indicator**	**CC**	**N**	**Indicator**	**CC**	**N**	**Indicator**	**CC**	**N**
1	Educational attainment, at least completed upper secondary, population 25+, total (%) (cumulative)	0.4 (0.24, 0.48)	88	Number of households 5 persons - Proportion over All households	-0.45 (-0.5, -0.36)	56	Poverty gap at $1.90 a day (2011 PPP) (%)	0.37 (0.21, 0.34)	92
2	Educational attainment, at least completed upper secondary, population 25+, female (%) (cumulative)	0.4 (0.25, 0.47)	88	Number of households 5 persons - Per capita	-0.41 (-0.46, -0.32)	56	Poverty headcount ratio at $5.50 a day (2011 PPP) (% of population)	0.36 (0.22, 0.37)	92
3	Educational attainment, at least completed upper secondary, population 25+, male (%) (cumulative)	0.39 (0.22, 0.47)	88	Number of households 6 persons and over - Per capita	-0.33 (-0.41, -0.22)	53	Crude death rate per 1000 population	0.35 (0.2, 0.37)	98
4	Number of households 5 persons - Proportion over All households	-0.34 (-0.42, -0.19)	56	Physicians (per 1,000 people)	0.32 (0.22, 0.37)	98	Life expectancy at birth (both sexes)	0.34 (0.2, 0.33)	98
5	Physicians (per 1,000 people)	0.33 (0.16, 0.41)	98	Prevalence of underweight, weight for age (% of children under 5)	-0.31 (-0.34, -0.21)	94	Poverty gap at $3.20 a day (2011 PPP) (% of population)	0.34 (0.22, 0.36)	92
6	Proportion of the population aged 65+	0.32 (0.2, 0.51)	98	Immunization, measles (% of children ages 12-23 months)	0.3 (0.22, 0.36)	98	Poverty headcount ratio at $1.90 a day (2011 PPP) (% of population)	0.34 (0.22, 0.36)	92
7	Number of households 5 persons - Per capita	-0.28 (-0.35, -0.1)	56	Number of households 6 persons and over - Proportion over All households	-0.29 (-0.37, -0.18)	53	Poverty gap at $5.50 a day (2011 PPP) (% of population)	0.33 (0.23, 0.37)	92

Table A (continued)

Rank	Pearson			Spearman			MIC		
	Indicator	CC	N	Indicator	CC	N	Indicator	CC	N
8	Proportion of the population aged 0-14	-0.27 (-0.39, -0.14)	98	Health expenditure, total (% of GDP)	0.28 (0.21, 0.35)	98	Poverty headcount ratio at $3.20 a day (2011 PPP) (% of population)	0.33 (0.22, 0.37)	92
9	Population living in slums (% of urban population)	-0.27 (-0.32, -0.13)	75	Urban population (% of total)	0.28 (0.2, 0.34)	98	Proportion of the population aged 0-4	0.32 (0.27, 0.38)	98
10	Number of households 6 persons and over - Proportion over All households	-0.26 (-0.34, -0.14)	53	Educational attainment, at least completed upper secondary, population 25+, total (%) (cumulative)	0.27 (0.2, 0.35)	88	Physicians (per 1,000 people)	0.31 (0.25, 0.37)	98
11	Number of households 6 persons and over - Per capita	-0.26 (-0.34, -0.09)	53	Immunization, DPT (% of children ages 12-23 months)	0.26 (0.2, 0.32)	98	GDP per capita, PPP (constant 2011 international $)	0.31 (0.2, 0.33)	98
12	Proportion of the population aged 0-4	-0.26 (-0.37, -0.13)	98	Low-birthweight babies (% of births)	-0.26 (-0.32, -0.19)	98	GDP per capita, PPP (current international $)	0.31 (0.2, 0.33)	98
13	Number of households 2 persons - Proportion over All households	0.25 (0.13, 0.36)	56	Educational attainment, at least completed upper secondary, population 25+, female (%) (cumulative)	0.26 (0.18, 0.33)	88	Probability of dying before age 5 (per 1000 live births)	0.31 (0.21, 0.33)	98
14	Poverty headcount ratio at $5.50 a day (2011 PPP) (% of population)	-0.25 (-0.33, -0.14)	92	Number of households 2 persons - Proportion over All households	0.25 (0.15, 0.32)	56	Low-birthweight babies (% of births)	0.3 (0.23, 0.39)	98
15	Number of households 1 person - Proportion over All households	0.25 (0.12, 0.33)	56	Educational attainment, at least completed upper secondary, population 25+, male (%) (cumulative)	0.23 (0.17, 0.31)	88	Poverty headcount ratio at national poverty lines (% of population)	0.3 (0.19, 0.32)	75

Table A (continued)

Rank	Pearson			Spearman			MIC		
	Indicator	CC	N	Indicator	CC	N	Indicator	CC	N
16	Poverty gap at $5.50 a day (2011 PPP) (% of population)	-0.24 (-0.3, -0.14)	92	Proportion of the population aged 0-4	-0.23 (-0.29, -0.15)	98	HDI	0.29 (0.23, 0.33)	98
17	Poverty headcount ratio at $3.20 a day (2011 PPP) (% of population)	-0.24 (-0.29, -0.14)	92	Lifetime risk of maternal death (1 in: rate varies by country)	0.23 (0.14, 0.29)	98	Prevalence of underweight, weight for age (% of children under 5)	0.29 (0.21, 0.34)	94
18	Urban population (% of total)	0.22 (0.11, 0.29)	98	Proportion of the population aged 0-14	-0.22 (-0.28, -0.14)	98	GDP per capita (1990 Int. GK$)	0.29 (0.2, 0.32)	97
19	Immunization, measles (% of children ages 12-23 months)	0.22 (0.03, 0.27)	98	Poverty headcount ratio at $3.20 a day (2011 PPP) (% of population)	-0.22 (-0.27, -0.13)	92	Number of doctors' consultations	0.29 (0.2, 0.45)	25
20	Poverty headcount ratio at national poverty lines (% of population)	-0.22 (-0.27, -0.11)	75	Proportion of the population aged 65+	0.22 (0.15, 0.29)	98	Health expenditure, total (% of GDP)	0.28 (0.21, 0.35)	98
21	Poverty gap at $3.20 a day (2011 PPP) (% of population)	-0.22 (-0.26, -0.13)	92	Poverty gap at $5.50 a day (2011 PPP) (% of population)	-0.22 (-0.27, -0.12)	92	Number of households 5 persons - Per capita	0.28 (0.25, 0.42)	56
22	Number of households 1 person - Per capita	0.21 (0.1, 0.29)	56	Poverty headcount ratio at $5.50 a day (2011 PPP) (% of population)	-0.22 (-0.27, -0.12)	92	Population growth rate (Average annual rate of population change (percentage))	0.27 (0.22, 0.35)	98
23	Poverty headcount ratio at $1.90 a day (2011 PPP) (% of population)	-0.21 (-0.25, -0.12)	92	Poverty gap at $3.20 a day (2011 PPP) (% of population)	-0.22 (-0.26, -0.12)	92	Immunization, measles (% of children ages 12-23 months)	0.27 (0.19, 0.32)	98

Table A (continued)

Rank	Pearson			Spearman			MIC		
	Indicator	CC	N	Indicator	CC	N	Indicator	CC	N
24	Number of households 2 persons - Per capita	0.21 (0.11 0.3)	56	Number of households 4 persons - Proportion over All households	-0.21 (-0.28 -0.11)	56	Urban population (% of total)	0.27 (0.22 0.32)	98
25	Prevalence of underweight, weight for age (% of children under 5)	-0.21 (-0.26 -0.08)	94	Exclusive breastfeeding (% of children under 6 months)	0.21 (0.11 0.27)	83	Number of households 3 persons - Per capita	0.27 (0.22 0.37)	56
26	Population growth rate (Average annual rate of population change (percentage))	-0.2 (-0.31 -0.13)	98	Poverty headcount ratio at $1.90 a day (2011 PPP) (% of population)	-0.21 (-0.26 -0.12)	92	Educational attainment, at least completed upper secondary, population 25+, total (%) (cumulative)	0.27 (0.22 0.36)	88
27	Lifetime risk of maternal death (1 in: rate varies by country)	0.2 (0.09 0.33)	98	HiB vaccination coverage	0.21 (0.14 0.24)	96	Mean age of child-bearing	0.27 (0.22 0.35)	98
28	HDI	0.2 (0.08 0.27)	98	Immunization, HepB3 (% of one-year-old children)	0.2 (0.13 0.25)	91	Lifetime risk of maternal death (1 in: rate varies by country)	0.27 (0.22 0.36)	98
29	Immunization, DPT (% of children ages 12-23 months)	0.19 (0.03 0.25)	98	Life expectancy at birth (both sexes)	0.19 (0.11 0.24)	98	Number of households All households - Per capita	0.27 (0.2 0.34)	61
30	Health expenditure, total (% of GDP)	0.19 (0.09 0.26)	98	Number of households 2 persons - Per capita	0.19 (0.09 0.27)	56	Employment to population ratio, 15+, female (%) (modeled ILO estimate)	0.26 (0.2 0.3)	98
31	GDP per capita (1990 Int. GK$)	0.19 (0.09 0.37)	97	Poverty headcount ratio at national poverty lines (% of population)	-0.19 (-0.23 -0.08)	75	Number of households 2 persons - Proportion over All households	0.26 (0.21 0.41)	56

Table A (continued)

Rank	Pearson			Spearman			MIC		
	**Indicator**	**CC**	**N**	**Indicator**	**CC**	**N**	**Indicator**	**CC**	**N**
32	Poverty gap at $1.90 a day (2011 PPP) (%)	-0.18 (-0.22 -0.1)	92	GDP per capita (1990 Int. GK$)	0.19 (0.1 0.24)	97	Number of households 5 persons - Proportion over All households	0.26 (0.23 0.38)	56
33	Exclusive breastfeeding (% of children under 6 months)	0.18 (0.04 0.23)	83	Probability of dying before age 5 (per 1000 live births)	-0.18 (-0.23 -0.1)	98	Number of households 4 persons - Proportion over All households	0.26 (0.21 0.37)	56
34	HiB vaccination coverage	0.18 (0.06 0.22)	96	Total fertility rate (live births per woman)	-0.18 (-0.24 -0.11)	98	Number of households 2 persons - Per capita	0.26 (0.23 0.38)	56
35	Income share held by lowest 10%	0.18 (0.03 0.3)	92	GDP per capita, current prices (Purchasing power parity; international dollars per capita)	0.18 (0.09 0.22)	98	Number of households 6 persons and over - Per capita	0.25 (0.21 0.41)	53
36	Income share held by lowest 20%	0.17 (0.03 0.3)	92	Prevalence of undernourishment (% of population)	-0.17 (-0.23 -0.06)	77	Immunization, DPT (% of children ages 12-23 months)	0.25 (0.21 0.35)	98
37	Total fertility rate (live births per woman)	-0.17 (-0.28 -0.06)	98	Population living in slums (% of urban population)	-0.17 (-0.21 -0.09)	75	GDP per capita, current prices (Purchasing power parity; international dollars per capita)	0.25 (0.2 0.32)	98
38	GDP per capita, current prices (Purchasing power parity; international dollars per capita)	0.17 (0.06 0.23)	98	Number of households 1 person - Proportion over All households	0.17 (0.08 0.24)	56	Immunization, HepB3 (% of one-year-old children)	0.25 (0.18 0.3)	91
39	Adjusted net enrollment rate, primary (% of primary school age children)	0.16 (0.05 0.22)	93	Population density (people per sq. km of land area)	-0.17 (-0.22 -0.09)	98	Total fertility rate (live births per woman)	0.25 (0.21 0.35)	98

Table A (continued)

Rank	Pearson			Spearman			MIC		
	Indicator	CC	N	Indicator	CC	N	Indicator	CC	N
40	Mean age of child-bearing	-0.15 (-0.24 -0.04)	98	HDI	0.16 (0.08 0.21)	98	Number of households 3 persons - Proportion over All households	0.25 (0.19 0.35)	56
41	Total population in a household, both sexes	-0.14 (-0.19 -0.01)	26	Poverty gap at $1.90 a day (2011 PPP) (%)	-0.15 (-0.21 -0.07)	92	Prevalence of undernourishment (% of population)	0.25 (0.2 0.34)	77
42	Income share held by highest 20%	-0.14 (-0.27 -0.0)	92	Adjusted net enrollment rate, primary (% of primary school age children)	0.15 (0.06 0.2)	93	Educational attainment, at least completed upper secondary, population 25+, male (%) (cumulative)	0.25 (0.21 0.34)	88
43	Life expectancy at birth (both sexes)	0.13 (0.02 0.23)	98	Number of households 1 person - Per capita	0.15 (0.06 0.23)	56	Number of households 6 persons and over - Proportion over All households	0.24 (0.21 0.42)	53
44	Low-birthweight babies (% of births)	-0.13 (-0.2 -0.06)	98	Number of households 4 persons - Per capita	-0.14 (-0.21 -0.03)	56	HiB vaccination coverage	0.24 (0.19 0.3)	96
45	Number of households 3 persons - Per capita	0.13 (0.04 0.22)	56	GDP per capita, PPP (constant 2011 international $)	0.14 (0.06 0.18)	98	Employment to population ratio, ages 15-24, female (%) (modeled ILO estimate)	0.24 (0.19 0.29)	98
46	Population density (people per sq. km of land area)	-0.1 (-0.12 -0.03)	98	Population growth rate (Average annual rate of population change (percentage))	-0.13 (-0.19 -0.07)	98	Unemployment, total (% of total labor force) (modeled ILO estimate)	0.24 (0.19 0.31)	98
47	GDP per capita, PPP (constant 2011 international $)	0.09 (0.02 0.19)	98	Total population in a household, both sexes	-0.12 (-0.2 -0.01)	26	Employment to population ratio, 15+, female (%) (national estimate)	0.24 (0.19 0.3)	85
48	Number of households All households - Per capita	0.09 (0.04 0.18)	61	GDP per capita, PPP (current international $)	0.12 (0.05 0.17)	98	Number of households 4 persons - Per capita	0.24 (0.22 0.36)	56
49	GDP per capita, PPP (current international $)	0.07 (0.01 0.15)	98	Income share held by highest 20%	-0.11 (-0.18 -0.04)	92	Proportion of the population aged 0-14	0.24 (0.22 0.35)	98

Table A (continued)

Rank	Pearson			Spearman			MIC		
	**Indicator**	**CC**	**N**	**Indicator**	**CC**	**N**	**Indicator**	**CC**	**N**
50	Average number of people per room in occupied housing unit	0.48 (-0.21 0.53)	12	Income share held by highest 10%	-0.11 (-0.18 -0.04)	92	Population living in slums (% of urban population)	0.24 (0.21 0.34)	75
51	Prevalence of undernourishment (% of population)	-0.15 (-0.2 0.03)	77	Employment to population ratio, ages 15-24, female (%) (modeled ILO estimate)	0.1 (0.04 0.16)	98	Population density (people per sq. km of land area)	0.24 (0.21 0.3)	98
52	Immunization, HepB3 (% of one-year-old children)	0.15 (-0.01 0.2)	91	Income share held by lowest 20%	0.1 (0.03 0.17)	92	Proportion of the population aged 65+	0.24 (0.21 0.32)	98
53	Income share held by highest 10%	-0.13 (-0.25 0.01)	92	Income share held by lowest 10%	0.1 (0.03 0.17)	92	Number of households 1 person - Per capita	0.23 (0.2 0.37)	56
54	Probability of dying before age 5 (per 1000 live births)	-0.11 (-0.21 0.02)	98	Employment to population ratio, 15+, female (%) (national estimate)	0.08 (0.01 0.14)	85	Exclusive breastfeeding (% of children under 6 months)	0.23 (0.2 0.34)	83
55	Employment to population ratio, 15+, female (%) (modeled ILO estimate)	-0.11 (-0.14 0.04)	98	Average number of people per room in occupied housing unit	0.29 (-0.15 0.36)	12	Employment to population ratio, ages 15-24, female (%) (national estimate)	0.23 (0.18 0.28)	80
56	Unemployment, total (% of total labor force) (modeled ILO estimate)	-0.11 (-0.15 0.02)	98	Number of doctors' consultations	-0.1 (-0.18 0.04)	25	Educational attainment, at least completed upper secondary, population 25+, female (%) (cumulative)	0.23 (0.23 0.36)	88
57	Number of households 4 persons - Proportion over All households	-0.09 (-0.2 0.04)	56	Unemployment, total (% of total labor force) (national estimate)	0.06 (-0.01 0.12)	98	Poverty gap at national poverty lines (%)	0.23 (0.22 0.4)	51

Table A (continued)

Rank	Pearson			Spearman			MIC		
	Indicator	CC	N	Indicator	CC	N	Indicator	CC	N
58	Number of households 3 persons - Proportion over All households	0.08 (-0.03 0.18)	56	Number of households 3 persons - Per capita	0.05 (-0.04 0.15)	56	Income share held by highest 10%	0.22 (0.17 0.28)	92
59	Number of doctors' consultations	0.07 (-0.11 0.25)	25	Number of households 3 persons - Proportion over All households	0.04 (-0.04 0.14)	56	Number of households 1 person - Proportion over All households	0.22 (0.17 0.32)	56
60	Poverty gap at national poverty lines (%)	-0.07 (-0.15 0.04)	51	Employment to population ratio, 15+, female (%) (modeled ILO estimate)	-0.03 (-0.08 0.05)	98	Adjusted net enrollment rate, primary (% of primary school age children)	0.22 (0.19 0.33)	93
61	Number of households 4 persons - Per capita	0.06 (-0.01 0.17)	56	Unemployment, total (% of total labor force) (modeled ILO estimate)	0.02 (-0.05 0.09)	98	Unemployment, total (% of total labor force) (national estimate)	0.21 (0.18 0.27)	98
62	Crude death rate per 1000 population	0.05 (-0.01 0.14)	98	Crude death rate per 1000 population	-0.02 (-0.04 0.07)	98	Income share held by lowest 10%	0.2 (0.18 0.3)	92
63	Employment to population ratio, 15+, female (%) (national estimate)	-0.05 (-0.09 0.09)	85	Mean age of child-bearing	-0.02 (-0.08 0.05)	98	Income share held by lowest 20%	0.19 (0.18 0.29)	92
64	Employment to population ratio, ages 15-24, female (%) (national estimate)	-0.03 (-0.07 0.13)	80	Poverty gap at national poverty lines (%)	0.02 (-0.07 0.11)	51	Income share held by highest 20%	0.19 (0.17 0.28)	92
65	Unemployment, total (% of total labor force) (national estimate)	0.02 (-0.07 0.1)	98	Employment to population ratio, ages 15-24, female (%) (national estimate)	0.01 (-0.05 0.08)	80	Total population in a household, both sexes	0.16 (0.16 0.39)	26
66	Employment to population ratio, ages 15-24, female (%) (modeled ILO estimate)	-0.01 (-0.04 0.17)	98	Number of households All households - Per capita	0.0 (-0.07 0.09)	61	Average number of people per room in occupied housing unit	0.09 (0.09 0.2)	12
