# Supplementary material for: Estimates of the basic reproduction number for rubella using seroprevalence data and indicator-based approaches
Source: PLoS Comput Biol. 2022 Mar 3;18(3):e1008858. doi: 10.1371/journal.pcbi.1008858 (PMC8893344; doi:10.1371/journal.pcbi.1008858)
Supplement: S1 Text — Fig A: Examples of two different splits to folds for the toy-case cross-validation scenario described above. The ‘x’ marks denote missing values. Table A: Number of studies with a valid indicator value in each fold for each of the 10 repetitions of the 4-fold cross validation experiment. The indicators order for the Simple Linear Regression case is the same as in Table A in S2 Table and the order of indicators subsets for the Random Forest case is the same as in the main text. The row labelled ‘Seed’ gives the seed numbers used for each of the 10 split to folds (omitted seed numbers corresponds to splits that did not have at least one study with a valid (non-missing) value in each fold for each one of the 66 indicators. (PDF) [file pcbi.1008858.s006.pdf]

Details of 4-fold cross-validation – Dealing with missing indicators in simple linear regression and random forest analyses

Pseudocode description

The following piece of pseudocode describes the practical details of the 4-fold cross-validation experiment and the calculation of the MSE metrics of the R_0 predictions from the linear regression and the random forest prediction algorithms against the values estimated using seroprevalence data as well as the calculation of the corresponding MSE metrics for the case where R_0 is calculated using the default method against the values estimated using study-specific seroprevalence data.

1. *RandomState* = 0. Initialize *RandomState* variable which is used as the seed for the pseudo-random split of the 98 studies to 4 folds (point 3.a)
2. *GoodStatesList* = [empty list]. Initialize *GoodStatesList* in which we keep the seeds that result in splits to 4 folds that are suitable for running the experiment (see point 3.b.i below)
3. while length(*GoodStatesList*) ≤ 10
   a. Split the 98 studies in 4 folds (24 or 25 studies in each) using *RandomState* as the seed
   b. Check whether for each one of the 66 indicators there is at least one study in each fold with a valid (non-missing) value of the indicator:
      i. If Yes: append *RandomState* to *GoodStatesList* and continue to the following step 3.c
      ii. If No: then increase the value of *RandomState* by 1 and jump to step 3.a

   Simple linear regression component:

   c. for *indicator, i,* ranging across the 66 indicators
      i. for *testfold, t,* ranging from 1 to 4
         A. Use those studies in *testfold* that have a valid value for *indicator i* as the test set (denote their number with N_{test}) and those studies in the other 3 folds that have a valid value for *indicator i* as the train set (denote their number with N_{train}). As a result of check in step 3.b.i it is guaranteed that $N_{test} \geq 1$ and $N_{train} \geq 3$. (A simple explanation of this is given in the toy-case example further below). The number of studies in the test and train set for each indicator in each fold and each different split to folds are given in the Table further below

B. Fit linear regression line to the N_{train} studies in the training set using the value R_0 of the basic reproduction number as estimated using seroprevalence data

C. Use the fitted line calculated in step 3.c.i.B to predict the basic reproduction number $\hat{R}_0$ for each of the N_{test} studies in the test set.

D. Compute the mean squared error for the studies in the test set, $MSE_{LR}(t,i)$ as

$$MSE_{LR}(t,i) = \sum_{j=1}^{N_{test}} \frac{\left(\hat{R}_{0_j} - R_{0_j}\right)^2}{N_{test}}$$

where $\hat{R}_{0_j}$ and R_{0_j} denote the basic reproduction number for the jth study of the test set predicted using linear regression line of step 3.c.i.B and estimated using seroprevalence data respectively.

E. Compute the mean squared error for the studies in the test set, $MSE_{LR,def}(t,i)$ as

$$MSE_{LR,def}(t,i) = \sum_{j=1}^{N_{test}} \frac{\left(\hat{R}_{0,def_j} - R_{0_j}\right)^2}{N_{test}}$$

where $\hat{R}_{0,def_j}$ and R_{0_j} denote the basic reproduction number for the jth study of the test set predicted using the default method and estimated using study-specific seroprevalence respectively

ii. Average the values of MSE over the 4 folds

A. Compute the mean of $MSE_{LR}(t, i)$ over the four *testfold* repetitions to get $MSE_{LR_{mean}}(i)$ (linear regression prediction):

$$MSE_{LR_{mean}}(i) = \sum_{t=1}^{4} MSE_{LR}(t,i)/4$$

B. Compute the mean of $MSE_{LR,def}(t, i)$ over the four *testfold* repetions to get $MSE_{LR,def_{mean}}(i)$ (regional average prediction):

$$MSE_{LR,def_{mean}}(i) = \sum_{t=1}^{4} \frac{MSE_{LR,def}(t,i)}{4}$$

Random forest component:

d. for *indicatorset*, I_k, k = 1, 2, 3, 4, 5 ranging across the 5 subsets of indicators described in the text (25 indicators that have no missing values, 43 indicators that have up to 10 missing values, etc.)

i. for *testfold, t,* ranging from 1 to 4

A. Use those studies in *testfold* that have valid values for all the indicators in *indicatorset*, I_k as the test set (denote their number with N_{test}) and those studies in the other 3 folds that have valid values for all the indicators in *indicatorset*, I_k as the training set (denote their number with N_{train}). Note that the check of

step 3.b.i guarantees neither that N_{test} > 0 nor that N_{train} > 0. A simple explanation of this is given in the toy-case example further below. The number of studies in the test and train set for each indicators set I_k in each fold and each different split to folds are given in the Table further below

B. If any of N_{test} or N_{train} is 0 raise a flag and stop the execution

C. Train a random forest with the N_{train} studies of the train set

D. Use the trained random forest of step 3.d.i.C to predict the basic reproduction number $\hat{R}_0$ for each of the N_{test} studies in the test set.

E. Compute the mean squared error for the studies in the test set $MSE_{RF}(t, I_k)$ as

$$MSE_{RF}(t, I_k) = \sum_{j=1}^{N_{test}} \frac{\left(\hat{R}_{0_j} - R_{0_j}\right)^2}{N_{test}}$$

where $\hat{R}_{0_j}$ and R_{0_j} denote the basic reproduction number for the jth study of the test set predicted using the random forest of step 3.d.i.C and estimated using seroprevalence data respectively

F. Compute the mean squared error for the studies in the test set $MSE_{RF,def}(t, I_k)$ as

$$MSE_{RF,def}(t, I_k) = \sum_{j=1}^{N_{test}} \frac{\left(\hat{R}_{0,def_j} - R_{0_j}\right)^2}{N_{test}}$$

where $\hat{R}_{0,def_j}$ and R_{0_j} is the basic reproduction number for the jth study of the test set predicted using the default method and estimated using study-specific seroprevalence data respectively

ii. Average the values of MSE over the 4 folds

A. Compute the mean of $MSE_{RF}(t, I_k)$ over the four *testfold* repetions to get $MSE_{RF_{mean}}(I_k)$ (random forest prediction):

$$MSE_{RF_{mean}}(I_k) = \sum_{t=1}^{4} MSE_{RF}(t, I_k)/4$$

B. Compute the mean of $MSE_{RF,def}(t, I_k)$ over the four *testfold* repetions to get $MSE_{RF,def_{mean}}(I_k)$ (regional average prediction):

$$MSE_{RF,def_{mean}}(I_k) = \sum_{t=1}^{4} \frac{MSE_{RF,def}(t, I_k)}{4}$$

e. Store values of $MSE_{LR_{mean}}(i)$ and $MSE_{LR,df_{mean}}(i)$ for each of the 66 indicators and $MSE_{RF_{mean}}(I_k)$ and $MSE_{RF,def_{mean}}(I_k)$ for each indicator set I_k (k = 1, .., 5)

f. Increase the value of *RandomState* by 1 and move to next iteration of while-loop in step 3

Missing values example

In the following we use a toy-case example to highlight the implications that missing indicator values have in the k-fold cross-validation experiments for the simple linear regression and the random forest prediction algorithms. We consider a simplistic scenario in which there are 9 data points (in our case studies) and 5 features (in our case socio-economic indicators). We take example case in which the missing values are distributed as follows:

- Indicator 1 has no missing values
- Indicators 2 and 3 are missing for Study 9
- Indicator 4 is missing in Studies 8 and 9
- Indicator 5 is missing in Studies 1, 5, 6, 7, and 8

We consider the case in which a 3-fold cross-validation is used. For a chosen split of the 9 studies to 3 folds, in order for cross-validation experiment to be feasible in the simple linear regression case, it is required that for every one of the indicators there exists at least one study in each one of the folds that has a valid value. Such an example of a split to folds where the simple linear regression cross-validation experiment is feasible for each one of the indicators is shown in Fig A (labelled as 'Example 1'). Furthermore, in this particular split to folds it can also be seen that there exists a study in each fold which has valid values for all indicators. This practically means that the cross-validation experiment is also feasible for the random forest case in which one predictor is fitted to all the indicators together.

In a second example of split to folds, shown in Fig A (labelled as 'Example 2'), it is also true that for every one of the indicators there is at least one study in each one of the folds which has a valid value. However, in the split of Example 2, it can be seen that Fold 3 does contain a study which has valid values for all indicators. Hence in this split the cross-validation experiment is feasible for the simple linear regression algorithm

on each of the indicators but not for the random forest algorithm which is trained and tested on all the indicators together. The random forest cross-validation experiment is however feasible in both splits of Fig A if we restrict the random forest training/testing to a subset of indicators, e.g. to indicators 1 to 4. As it can be seen in Fig A if we restrict the random forest to train/testing using indicators 1 to 4, then in example 2 split 1 has studies 1, 2 and 4 with full values, and the same for studies 3 and 7 in split 2 and studies 5 and 6 in split 3.

Fig A: Examples of two different splits to folds for the toy-case cross-validation scenario described above. The 'x' marks denote missing values

Example 1

Fold	Study	Indicator				
		1	2	3	4	5
1	4					
	9		x	x	x	
	1					x
2	3					
	8				x	x
	6					x
3	5					x
	2					
	7					x

Example 2

Fold	Study	Indicator				
		1	2	3	4	5
1	4					
	2					
	1					x
2	7					x
	8				x	x
	3					
3	5					x
	9		x	x	x	
	6					x

Overall, as can be seen in Fig A, in the example considered here there are only three studies that have valid values for all indicators so only a few splits to folds have exactly one of those 3 studies in each fold. By using a smaller set of indicators this condition is relaxed. For instance, there are 7 studies that have valid values for the subset containing indicators 1 to 4. Consequently, the cross-validation experiment is feasible for the random forest in more splits to folds.

As is shown in Table B in S2 Table, in the actual indicator data that we use for the results presented in this work, there is no study having a valid value for all indicators. Hence, in order to make possible the random forest cross-validation experiment

feasible we chose to run it on subsets of indicators as described in the main text. As can be seen in Table A, for the 5 subsets of indicators described in the main text it was the case that in each of the 10 splits to folds chosen for the simple linear regression experiment there was at least one study in each fold which had valid values for all indicators in the chosen indicators subset. This can be seen in Table A.

Table A: Number of studies with a valid indicator value in each fold for each of the 10 repetitions of the 4-fold cross validation experiment. The indicators order for the Simple Linear Regression case is the same as in Table A in S2 Table and the order of indicators subsets for the Random Forest case is the same as in the main text. The row labelled ‘Seed’ gives the seed numbers used for each of the 10 split to folds (omitted seed numbers corresponds to splits that did not have at least one study with a valid (non-missing) value in each fold for each one of the 66 indicators.

**Split**	**1**				**2**				**3**				**4**				**5**				**6**				**7**				**8**				**9**				**10**			
**Seed**	**0**				**8**				**9**				**10**				**11**				**12**				**13**				**14**				**16**				**17**			
**Fold**	**1**	**2**	**3**	**4**	**1**	**2**	**3**	**4**	**1**	**2**	**3**	**4**	**1**	**2**	**3**	**4**	**1**	**2**	**3**	**4**	**1**	**2**	**3**	**4**	**1**	**2**	**3**	**4**	**1**	**2**	**3**	**4**	**1**	**2**	**3**	**4**	**1**	**2**	**3**	**4**
**Indicator**	**Simple Linear Regression**																																							
**1**	25	25	24	24	25	25	24	24	25	25	24	24	25	25	24	24	25	25	24	24	25	25	24	24	25	25	24	24	25	25	24	24	25	25	24	24	25	25	24	24
**2**	25	25	24	24	25	25	24	24	25	25	24	24	25	25	24	24	25	25	24	24	25	25	24	24	25	25	24	24	25	25	24	24	25	25	24	24	25	25	24	24
**3**	25	25	24	24	25	25	24	24	25	25	24	24	25	25	24	24	25	25	24	24	25	25	24	24	25	25	24	24	25	25	24	24	25	25	24	24	25	25	24	24
**4**	25	25	24	24	25	25	24	24	25	25	24	24	25	25	24	24	25	25	24	24	25	25	24	24	25	25	24	24	25	25	24	24	25	25	24	24	25	25	24	24
**5**	25	25	24	24	25	25	24	24	25	25	24	24	25	25	24	24	25	25	24	24	25	25	24	24	25	25	24	24	25	25	24	24	25	25	24	24	25	25	24	24
**6**	25	25	24	24	25	25	24	24	25	25	24	24	25	25	24	24	25	25	24	24	25	25	24	24	25	25	24	24	25	25	24	24	25	25	24	24	25	25	24	24
**7**	25	25	24	24	25	25	24	24	25	25	24	24	25	25	24	24	25	25	24	24	25	25	24	24	25	25	24	24	25	25	24	24	25	25	24	24	25	25	24	24
**8**	25	25	24	24	25	25	24	24	25	25	24	24	25	25	24	24	25	25	24	24	25	25	24	24	25	25	24	24	25	25	24	24	25	25	24	24	25	25	24	24
**9**	25	25	24	24	25	25	24	24	25	25	24	24	25	25	24	24	25	25	24	24	25	25	24	24	25	25	24	24	25	25	24	24	25	25	24	24	25	25	24	24
**10**	25	25	24	24	25	25	24	24	25	25	24	24	25	25	24	24	25	25	24	24	25	25	24	24	25	25	24	24	25	25	24	24	25	25	24	24	25	25	24	24
**11**	25	25	24	24	25	25	24	24	25	25	24	24	25	25	24	24	25	25	24	24	25	25	24	24	25	25	24	24	25	25	24	24	25	25	24	24	25	25	24	24
**12**	25	25	24	24	25	25	24	24	25	25	24	24	25	25	24	24	25	25	24	24	25	25	24	24	25	25	24	24	25	25	24	24	25	25	24	24	25	25	24	24
**13**	23	23	23	23	24	22	24	22	23	23	22	24	22	24	22	24	23	24	22	23	24	24	23	21	24	25	24	19	25	24	22	21	22	25	22	23	24	23	23	22
**14**	23	23	23	23	24	22	24	22	23	23	22	24	22	24	22	24	23	24	22	23	24	24	23	21	24	25	24	19	25	24	22	21	22	25	22	23	24	23	23	22
**15**	23	23	23	23	24	22	24	22	23	23	22	24	22	24	22	24	23	24	22	23	24	24	23	21	24	25	24	19	25	24	22	21	22	25	22	23	24	23	23	22
**16**	23	23	23	23	24	22	24	22	23	23	22	24	22	24	22	24	23	24	22	23	24	24	23	21	24	25	24	19	25	24	22	21	22	25	22	23	24	23	23	22
**17**	23	23	23	23	24	22	24	22	23	23	22	24	22	24	22	24	23	24	22	23	24	24	23	21	24	25	24	19	25	24	22	21	22	25	22	23	24	23	23	22
**18**	23	23	23	23	24	22	24	22	23	23	22	24	22	24	22	24	23	24	22	23	24	24	23	21	24	25	24	19	25	24	22	21	22	25	22	23	24	23	23	22
**19**	23	23	23	23	24	22	24	22	23	23	22	24	22	24	22	24	23	24	22	23	24	24	23	21	24	25	24	19	25	24	22	21	22	25	22	23	24	23	23	22
**20**	15	13	10	13	11	13	12	15	14	12	9	16	11	15	11	14	14	14	11	12	16	6	15	14	12	15	13	11	12	14	13	12	13	14	10	14	12	14	12	13

**21**	23	23	23	23	24	22	24	22	23	23	22	24	22	24	22	24	23	24	22	23	24	24	23	21	24	25	24	19	25	24	22	21	22	25	22	23	24	23	23	22
**22**	23	23	23	23	24	22	24	22	23	23	22	24	22	24	22	24	23	24	22	23	24	24	23	21	24	25	24	19	25	24	22	21	22	25	22	23	24	23	23	22
**23**	23	23	23	23	24	22	24	22	23	23	22	24	22	24	22	24	23	24	22	23	24	24	23	21	24	25	24	19	25	24	22	21	22	25	22	23	24	23	23	22
**24**	19	18	20	18	18	20	17	20	22	19	14	20	16	22	18	19	21	17	20	17	20	17	19	19	20	20	21	14	18	20	19	18	19	20	16	20	16	21	19	19
**25**	25	25	24	24	25	25	24	24	25	25	24	24	25	25	24	24	25	25	24	24	25	25	24	24	25	25	24	24	25	25	24	24	25	25	24	24	25	25	24	24
**26**	25	25	24	24	25	25	24	24	25	25	24	24	25	25	24	24	25	25	24	24	25	25	24	24	25	25	24	24	25	25	24	24	25	25	24	24	25	25	24	24
**27**	25	25	24	24	25	25	24	24	25	25	24	24	25	25	24	24	25	25	24	24	25	25	24	24	25	25	24	24	25	25	24	24	25	25	24	24	25	25	24	24
**28**	25	25	24	24	25	25	24	24	25	25	24	24	25	25	24	24	25	25	24	24	25	25	24	24	25	25	24	24	25	25	24	24	25	25	24	24	25	25	24	24
**29**	24	25	24	24	25	25	24	23	25	24	24	24	25	25	23	24	25	25	23	24	25	25	24	23	25	25	23	24	25	24	24	24	25	25	24	23	25	25	24	23
**30**	24	25	22	22	24	21	24	24	24	24	22	23	24	24	23	22	23	25	24	21	25	24	22	22	25	24	22	22	23	25	22	23	25	24	22	22	23	22	24	24
**31**	24	23	22	19	21	21	23	23	21	23	22	22	23	23	23	19	23	23	22	20	21	24	23	20	19	21	24	24	22	22	21	23	23	21	22	22	21	22	23	22
**32**	24	23	22	19	21	21	23	23	21	23	22	22	23	23	23	19	23	23	22	20	21	24	23	20	19	21	24	24	22	22	21	23	23	21	22	22	21	22	23	22
**33**	24	23	22	19	21	21	23	23	21	23	22	22	23	23	23	19	23	23	22	20	21	24	23	20	19	21	24	24	22	22	21	23	23	21	22	22	21	22	23	22
**34**	25	25	24	24	25	25	24	24	25	25	24	24	25	25	24	24	25	25	24	24	25	25	24	24	25	25	24	24	25	25	24	24	25	25	24	24	25	25	24	24
**35**	25	25	24	24	25	25	24	24	25	25	24	24	25	25	24	24	25	25	24	24	25	25	24	24	25	25	24	24	25	25	24	24	25	25	24	24	25	25	24	24
**36**	25	25	24	24	25	25	24	24	25	25	24	24	25	25	24	24	25	25	24	24	25	25	24	24	25	25	24	24	25	25	24	24	25	25	24	24	25	25	24	24
**37**	24	24	19	18	18	22	23	22	18	24	21	22	20	24	21	20	21	23	23	18	22	22	21	20	23	20	20	22	24	21	22	18	20	21	24	20	21	23	23	18
**38**	25	25	24	24	25	25	24	24	25	25	24	24	25	25	24	24	25	25	24	24	25	25	24	24	25	25	24	24	25	25	24	24	25	25	24	24	25	25	24	24
**39**	21	24	19	16	16	22	23	19	17	21	21	21	20	22	19	19	19	23	21	17	20	22	20	18	23	19	19	19	24	20	20	16	19	20	23	18	21	21	22	16
**40**	25	25	24	24	25	25	24	24	25	25	24	24	25	25	24	24	25	25	24	24	25	25	24	24	25	25	24	24	25	25	24	24	25	25	24	24	25	25	24	24
**41**	20	21	18	18	19	20	19	19	20	20	18	19	20	22	16	19	18	21	21	17	21	19	19	18	22	19	19	17	19	19	20	19	21	21	16	19	19	19	19	20
**42**	22	25	24	23	24	25	22	23	24	24	23	23	22	25	24	23	25	23	23	23	24	25	22	23	25	24	23	22	23	25	23	23	24	24	24	22	24	24	23	23
**43**	25	25	24	24	25	25	24	24	25	25	24	24	25	25	24	24	25	25	24	24	25	25	24	24	25	25	24	24	25	25	24	24	25	25	24	24	25	25	24	24
**44**	25	25	24	24	25	25	24	24	25	25	24	24	25	25	24	24	25	25	24	24	25	25	24	24	25	25	24	24	25	25	24	24	25	25	24	24	25	25	24	24
**45**	21	23	23	24	22	24	22	23	24	24	21	22	22	25	21	23	23	22	23	23	24	22	22	23	25	22	24	20	23	23	23	22	23	23	22	23	24	24	20	23
**46**	25	25	24	24	25	25	24	24	25	25	24	24	25	25	24	24	25	25	24	24	25	25	24	24	25	25	24	24	25	25	24	24	25	25	24	24	25	25	24	24
**47**	20	22	21	20	22	20	19	22	23	22	18	20	21	22	20	20	22	21	22	18	22	22	19	20	23	21	21	18	19	22	20	22	22	22	18	21	20	21	20	22
**48**	25	25	24	24	25	25	24	24	25	25	24	24	25	25	24	24	25	25	24	24	25	25	24	24	25	25	24	24	25	25	24	24	25	25	24	24	25	25	24	24
**49**	8	7	6	4	5	8	8	4	6	4	9	6	5	6	8	6	4	6	8	7	4	8	8	5	5	6	8	6	9	6	4	6	5	6	7	7	6	4	12	3

**50**	25	25	22	24	24	25	24	23	24	25	23	24	25	25	24	22	25	25	23	23	25	24	24	23	24	25	23	24	25	25	23	23	25	24	24	23	24	24	24	24
**51**	4	3	2	3	3	2	5	2	3	3	4	2	4	2	4	2	3	2	5	2	3	4	2	3	4	2	3	3	4	2	3	3	4	2	4	2	2	5	3	2
**52**	18	20	19	18	21	19	17	18	21	18	17	19	21	20	16	18	19	19	20	17	19	19	19	18	21	20	18	16	18	19	19	19	20	21	14	20	19	17	18	21
**53**	14	19	17	11	12	13	19	17	15	14	16	16	14	16	15	16	13	18	16	14	16	18	15	12	18	14	16	13	16	15	13	17	12	17	19	13	13	16	20	12
**54**	13	17	15	11	10	11	18	17	13	13	15	15	13	14	13	16	13	18	12	13	15	16	15	10	16	14	15	11	14	15	12	15	11	17	16	12	11	15	20	10
**55**	13	17	15	11	10	11	18	17	13	13	15	15	13	14	13	16	13	18	12	13	15	16	15	10	16	14	15	11	14	15	12	15	11	17	16	12	11	15	20	10
**56**	13	17	15	11	10	11	18	17	13	13	15	15	13	14	13	16	13	18	12	13	15	16	15	10	16	14	15	11	14	15	12	15	11	17	16	12	11	15	20	10
**57**	13	17	15	11	10	11	18	17	13	13	15	15	13	14	13	16	13	18	12	13	15	16	15	10	16	14	15	11	14	15	12	15	11	17	16	12	11	15	20	10
**58**	13	17	15	11	10	11	18	17	13	13	15	15	13	14	13	16	13	18	12	13	15	16	15	10	16	14	15	11	14	15	12	15	11	17	16	12	11	15	20	10
**59**	13	17	15	11	10	11	18	17	13	13	15	15	13	14	13	16	13	18	12	13	15	16	15	10	16	14	15	11	14	15	12	15	11	17	16	12	11	15	20	10
**60**	13	17	15	11	10	11	18	17	13	13	15	15	13	14	13	16	13	18	12	13	15	16	15	10	16	14	15	11	14	15	12	15	11	17	16	12	11	15	20	10
**61**	13	17	15	11	10	11	18	17	13	13	15	15	13	14	13	16	13	18	12	13	15	16	15	10	16	14	15	11	14	15	12	15	11	17	16	12	11	15	20	10
**62**	13	17	15	11	10	11	18	17	13	13	15	15	13	14	13	16	13	18	12	13	15	16	15	10	16	14	15	11	14	15	12	15	11	17	16	12	11	15	20	10
**63**	13	17	15	11	10	11	18	17	13	13	15	15	13	14	13	16	13	18	12	13	15	16	15	10	16	14	15	11	14	15	12	15	11	17	16	12	11	15	20	10
**64**	13	15	15	10	9	11	16	17	12	13	13	15	11	14	12	16	13	16	11	13	14	14	15	10	15	13	15	10	14	14	11	14	11	14	16	12	10	15	20	8
**65**	13	15	15	10	9	11	16	17	12	13	13	15	11	14	12	16	13	16	11	13	14	14	15	10	15	13	15	10	14	14	11	14	11	14	16	12	10	15	20	8
**66**	6	7	9	4	4	8	7	7	6	4	9	7	2	7	8	9	4	10	4	8	7	8	6	5	8	7	8	3	6	9	4	7	5	6	9	6	5	6	12	3
**Indicator Subset**	**Random Forest**																																							
**1**	25	25	24	24	25	25	24	24	25	25	24	24	25	25	24	24	25	25	24	24	25	25	24	24	25	25	24	24	25	25	24	24	25	25	24	24	25	25	24	24
**2**	16	19	18	16	16	15	20	18	17	18	15	19	17	22	16	14	18	19	17	15	19	19	18	13	17	18	20	14	18	18	16	17	18	17	17	17	17	16	18	18
**3**	13	17	13	9	10	11	17	14	12	14	11	15	14	16	13	9	13	16	15	8	14	17	14	7	15	12	16	9	15	15	11	11	12	14	13	13	12	13	16	11
**4**	9	12	8	3	4	5	12	11	8	8	6	10	9	8	9	6	8	9	10	5	9	8	10	5	9	7	11	5	10	9	4	9	8	8	7	9	5	7	14	6
**5**	6	9	3	2	1	1	8	10	5	4	4	7	6	4	5	5	6	8	4	2	8	2	7	3	5	5	7	3	5	6	3	6	5	6	3	6	3	5	8	4
