# Supplementary material for: Estimates of the basic reproduction number for rubella using seroprevalence data and indicator-based approaches
Source: PLoS Comput Biol. 2022 Mar 3;18(3):e1008858. doi: 10.1371/journal.pcbi.1008858 (PMC8893344; doi:10.1371/journal.pcbi.1008858)
Supplement: S2 Text — Table A: Presence or absence of the two studies with highest value of R0 from non-imputed linear regression 4-fold cross validation experiments. Table B: Presence or absence of the two studies with highest value of R0 from non-imputed random forest 4-fold cross validation experiments. Fig A: Similar to Fig 3 of the main text but with the two studies (‘Czech Republic, <1967’ and ‘Chile (rural), 1967–68’) excluded. Mean value (blue and orange dots) and minimum-maximum value range (blue and orange line) of the MSE of the predicted R0 over ten 4-fold cross validation splits. Fig B: Similar to Fig 4 of the main text but with the two studies (‘Czech Republic, <1967’ and ‘Chile (rural), 1967–68’) excluded. Mean value (blue and orange dots) and minimum-maximum value range (blue and orange line) of the MSE of the predicted R0 over ten 4-fold cross validation splits. (PDF) [file pcbi.1008858.s007.pdf]

Effect of studies with high R_0 values on the performance of simple linear regression and Random Forest prediction

In this section we explain in detail how the MSE variation of simple linear regression on different indicators can be directly traced to particular combinations of missing indicator values and the value of R_0 as calculated using seroprevalence data. Consequently, we argue that this variation in the MSE should not be interpreted as increased predictive power of some indicators over others.

More specifically, we show that those indicators that have a missing value for either or both of the high seroprevalence-estimated R_0 value studies ('Czech Republic, <1967' and 'Chile (rural), 1967-68' with R_0 value equal to 19.97 and 16.53 respectively) are associated with worse MSE performance. In simple terms, the trained model prediction output cannot reach those high R_0 values in either of the two regression methods that we consider (simple linear regression and random forest). Hence the indicators which have a missing value for both those studies have an advantage in terms of the prediction MSE.

Contrary to that, that indicators which have a valid value for both those studies are associated with a higher MSE. Furthermore, this increase in the MSE is further exacerbated for those indicators which have very few valid values overall, as the higher error due to the two high R_0 studies is averaged over a smaller number of total studies. Hence, indicators with a valid value for the 'Czech Republic, <1967' and 'Chile (rural), 1967-68' studies but with valid values for a few studies overall are associated with the highest MSE.

Starting from the simple linear regression case, as can be seen in Table A (which also has a version sorted by MSE value for presentation clarity), the best performing

indicator (‘Poverty gap at national poverty lines (%)’ –listed in line 20 of Table A with MSE value equal to 2.73) is the only indicator having a missing value for both those studies (this indicator also has a missing value for the third highest R_0 study which is ‘East Germany, 1990’ with R_0 equal to 10.19).

Furthermore, 4 out of the 6 indicators that have a missing value in exactly one of those two studies, are directly following in terms of MSE performance (‘Adjusted net enrollment rate, primary (% of primary school age children)’, ‘Prevalence of undernourishment (% of population)’, ‘Exclusive breastfeeding (% of children under 6 months)’ and ‘Population living in slums (% of urban population)’ listed in line numbers 30, 41, 47 and 52 of Table A with MSE values equal to 5.35, 5.5, 5.36 and 5.39 respectively). Those 4 indicators have valid values for at least 75 studies in total. The remaining two indicators which have a missing value in exactly one of the two high R_0 studies (‘Average number of people per room in occupied housing unit’ and ‘Total population in a household, both sexes’) have valid values for only 12 and 26 studies in total (see Table A). As was described above, this practically means that the higher error due to high R_0 study is averaged over a much smaller number of total studies and, consequently, the MSE for those two indicators is much higher (33.32 and 17.4 respectively).

Considering the indicators which have a valid value for both of the high R_0 studies (‘Czech Republic, <1967’ and ‘Chile (rural), 1967-68’) the aforementioned effects result in a clear three-way division in terms of performance: A total of 45 indicators have valid values for 75 up to 98 studies and the MSE associated with them is clustered between the levels of 7.41 and 9.57 (those are the indicators listed in Table A in positions 1-19, 21-29, 31-40, 42-46, 48 and 50). A second group contains 13 indicators which have valid values for 53 up to 61 studies and the MSE associated with them is clustered between the levels of 10.15 and 12.26 (those are the indicators listed in Table A in positions 53-65). A third group contains one indicator (‘Number of doctors'

consultations') which has valid values for only 25 studies in total and for which the MSE raises to a value of 26.93

A similar effect can be seen in the random forest performance results regarding the indicator subsets that are used to train and the random forest (a tabular summary is included in Table B). In the cross validation experiment conducted using only the 25 indicators that have no missing values, both the high R0 studies are present and the overall error has the value of 9.87. In the experiments conducted using the indicators that have up to 10, 20 and 40 missing values only one of the two high R0 studies is present and the overall error is lower. However, as the number of studies involved in the experiment reduces from 69 to 52 and then 32 as we allow indicators with progressively more missing values, the overall error progressively increases (8.19, 9.6 and 13.8 respectively). Finally, none of the two high R0 studies is included in the experiment conducted with the 20 indicators having up to 70 missing values and the overall error in the case attains the lowest value of 6.88.

None of those effects can be seen in the imputed results (Fig 4 of the main text), with the exception of a small increase in the max range of the error value for the indicators 'Number of doctors' consultations' and 'Average number of people per room in occupied housing unit' indicators which are the ones with the fewest valid values overall (and hence the most imputed values).

Finally, in Figs A, B we plot the MSE values calculated in the same way as for Figs 3, 4 of the main text but when the cross-validation experiment is run after excluding the 'Czech Republic, <1967' and 'Chile (rural), 1967-68' studies. In this case the highest value of R_0 as estimated from seroprevalence data is for the 'East Germany, 1990' study (R_0=10.19). Most of the MSE variation can be seen in Fig A to be lost in this case. The only variation is a small decrease in the MSE value for the 5 indicators which have a missing value for the East Germany study ('Poverty gap at national poverty

lines (%)’, ‘Poverty headcount ratio at national poverty lines (% of population)’, ‘Prevalence of undernourishment (% of population)’, ‘Exclusive breastfeeding (% of children under 6 months)’ and ‘Population living in slums (% of urban population)’) and an increase in the MSE for the indicators with the fewest overall valid values (‘Average number of people per room in occupied housing unit’ and ‘Number of doctors' consultations’). Both those effects follow exactly the same pattern described above.

Table A: Presence or absence of the two studies with highest value of R_0 from non-imputed linear regression 4-fold cross validation experiments.

		Chile (rural), 1967-68	Czech Republic, <1967	Average MSE over 10 repetitions	Number of studies with valid values
1	Proportion of the population aged 0-4	+	+	7.81	98
2	Proportion of the population aged 0-14	+	+	7.77	98
3	Proportion of the population aged 65+	+	+	7.51	98
4	Lifetime risk of maternal death (1 in: rate varies by country)	+	+	7.96	98
5	Probability of dying before age 5 (per 1000 live births)	+	+	8.12	98
6	Crude death rate per 1000 population	+	+	8.06	98
7	Life expectancy at birth (both sexes)	+	+	8.04	98
8	Total fertility rate (live births per woman)	+	+	8.04	98
9	Mean age of child-bearing	+	+	8.32	98
10	Population growth rate (Average annual rate of population change (percentage))	+	+	7.98	98
11	Population density (people per sq. km of land area)	+	+	8.11	98
12	Urban population (% of total)	+	+	7.76	98
13	Income share held by highest 10%	+	+	9.21	92
14	Income share held by highest 20%	+	+	9.2	92
15	Income share held by lowest 10%	+	+	9.08	92
16	Income share held by lowest 20%	+	+	9.03	92
17	Poverty gap at $1.90 a day (2011 PPP) (%)	+	+	8.27	92
18	Poverty gap at $3.20 a day (2011 PPP) (% of population)	+	+	8.22	92
19	Poverty gap at $5.50 a day (2011 PPP) (% of population)	+	+	8.19	92
20	Poverty gap at national poverty lines (%)	-	-	2.73	51
21	Poverty headcount ratio at $1.90 a day (2011 PPP) (% of population)	+	+	8.24	92
22	Poverty headcount ratio at $3.20 a day (2011 PPP) (% of population)	+	+	8.21	92
23	Poverty headcount ratio at $5.50 a day (2011 PPP) (% of population)	+	+	8.24	92
24	Poverty headcount ratio at national poverty lines (% of population)	+	+	9.46	75
25	GDP per capita, PPP (constant 2011 international $)	+	+	8.06	98
26	GDP per capita, PPP (current international $)	+	+	8.09	98
27	HDI	+	+	7.84	98
28	GDP per capita, current prices (Purchasing power parity; international dollars per capita)	+	+	8.07	98
29	GDP per capita (1990 Int. GK$)	+	+	7.89	97
30	Adjusted net enrollment rate, primary (% of primary school age children)	+	-	5.35	93
31	Educational attainment, at least completed upper secondary, population 25+, female (%) (cumulative)	+	+	7.72	88
32	Educational attainment, at least completed upper secondary, population 25+, male (%) (cumulative)	+	+	7.76	88

33	Educational attainment, at least completed upper secondary, population 25+, total (%) (cumulative)	+	+	7.69	88
34	Unemployment, total (% of total labor force) (modeled ILO estimate)	+	+	8.1	98
35	Unemployment, total (% of total labor force) (national estimate)	+	+	8.26	98
36	Employment to population ratio, 15+, female (%) (modeled ILO estimate)	+	+	8	98
37	Employment to population ratio, 15+, female (%) (national estimate)	+	+	9.04	85
38	Employment to population ratio, ages 15-24, female (%) (modeled ILO estimate)	+	+	8.11	98
39	Employment to population ratio, ages 15-24, female (%) (national estimate)	+	+	9.57	80
40	Low-birthweight babies (% of births)	+	+	8.06	98
41	Prevalence of undernourishment (% of population)	+	-	5.5	77
42	Prevalence of underweight, weight for age (% of children under 5)	+	+	8.22	94
43	Health expenditure, total (% of GDP)	+	+	7.83	98
44	Immunization, DPT (% of children ages 12-23 months)	+	+	8	98
45	Immunization, HepB3 (% of one-year-old children)	+	+	8.57	91
46	Immunization, measles (% of children ages 12-23 months)	+	+	7.91	98
47	Exclusive breastfeeding (% of children under 6 months)	+	-	5.36	83
48	Physicians (per 1,000 people)	+	+	7.41	98
49	Number of doctors' consultations	+	+	26.21	25
50	HiB vaccination coverage	+	+	8.07	96
51	Average number of people per room in occupied housing unit	-	+	33.32	12
52	Population living in slums (% of urban population)	+	-	5.39	75
53	Number of households All households - Per capita	+	+	11.51	61
54	Number of households 1 person - Per capita	+	+	11.82	56
55	Number of households 1 person - Proportion over All households	+	+	11.89	56
56	Number of households 2 persons - Per capita	+	+	11.66	56
57	Number of households 2 persons - Proportion over All households	+	+	11.59	56
58	Number of households 3 persons - Per capita	+	+	11.94	56
59	Number of households 3 persons - Proportion over All households	+	+	12.09	56
60	Number of households 4 persons - Per capita	+	+	12.19	56
61	Number of households 4 persons - Proportion over All households	+	+	12.26	56
62	Number of households 5 persons - Per capita	+	+	11.64	56
63	Number of households 5 persons - Proportion over All households	+	+	11.15	56
64	Number of households 6 persons and over - Per capita	+	+	12.07	53
65	Number of households 6 persons and over - Proportion over All households	+	+	11.96	53
66	Total population in a household, both sexes	-	+	17.4	26

Table A (sorted by average MSE): Presence or absence of the two studies with highest value of R_0 from non-imputed linear regression 4-fold cross validation experiments.

		Chile (rural), 1967-68	Czech Republic, <1967	Average MSE over 10 repetitions	Number of studies with valid values
20	Poverty gap at national poverty lines (%)	-	-	2.73	51
30	Adjusted net enrollment rate, primary (% of primary school age children)	+	-	5.35	93
47	Exclusive breastfeeding (% of children under 6 months)	+	-	5.36	83
52	Population living in slums (% of urban population)	+	-	5.39	75
41	Prevalence of undernourishment (% of population)	+	-	5.5	77
48	Physicians (per 1,000 people)	+	+	7.41	98
3	Proportion of the population aged 65+	+	+	7.51	98
33	Educational attainment, at least completed upper secondary, population 25+, total (%) (cumulative)	+	+	7.69	88
31	Educational attainment, at least completed upper secondary, population 25+, female (%) (cumulative)	+	+	7.72	88
12	Urban population (% of total)	+	+	7.76	98
32	Educational attainment, at least completed upper secondary, population 25+, male (%) (cumulative)	+	+	7.76	88
2	Proportion of the population aged 0-14	+	+	7.77	98
1	Proportion of the population aged 0-4	+	+	7.81	98
43	Health expenditure, total (% of GDP)	+	+	7.83	98
27	HDI	+	+	7.84	98
29	GDP per capita (1990 Int. GK$)	+	+	7.89	97
46	Immunization, measles (% of children ages 12-23 months)	+	+	7.91	98
4	Lifetime risk of maternal death (1 in: rate varies by country)	+	+	7.96	98
10	Population growth rate (Average annual rate of population change (percentage))	+	+	7.98	98
36	Employment to population ratio, 15+, female (%) (modeled ILO estimate)	+	+	8	98
44	Immunization, DPT (% of children ages 12-23 months)	+	+	8	98
7	Life expectancy at birth (both sexes)	+	+	8.04	98
8	Total fertility rate (live births per woman)	+	+	8.04	98
6	Crude death rate per 1000 population	+	+	8.06	98
25	GDP per capita, PPP (constant 2011 international $)	+	+	8.06	98
40	Low-birthweight babies (% of births)	+	+	8.06	98
28	GDP per capita, current prices (Purchasing power parity; international dollars per capita)	+	+	8.07	98
50	HiB vaccination coverage	+	+	8.07	96
26	GDP per capita, PPP (current international $)	+	+	8.09	98
34	Unemployment, total (% of total labor force) (modeled ILO estimate)	+	+	8.1	98
11	Population density (people per sq. km of land area)	+	+	8.11	98
38	Employment to population ratio, ages 15-24, female (%) (modeled ILO estimate)	+	+	8.11	98

5	Probability of dying before age 5 (per 1000 live births)	+	+	8.12	98
19	Poverty gap at $5.50 a day (2011 PPP) (% of population)	+	+	8.19	92
22	Poverty headcount ratio at $3.20 a day (2011 PPP) (% of population)	+	+	8.21	92
18	Poverty gap at $3.20 a day (2011 PPP) (% of population)	+	+	8.22	92
42	Prevalence of underweight, weight for age (% of children under 5)	+	+	8.22	94
21	Poverty headcount ratio at $1.90 a day (2011 PPP) (% of population)	+	+	8.24	92
23	Poverty headcount ratio at $5.50 a day (2011 PPP) (% of population)	+	+	8.24	92
35	Unemployment, total (% of total labor force) (national estimate)	+	+	8.26	98
17	Poverty gap at $1.90 a day (2011 PPP) (%)	+	+	8.27	92
9	Mean age of child-bearing	+	+	8.32	98
45	Immunization, HepB3 (% of one-year-old children)	+	+	8.57	91
16	Income share held by lowest 20%	+	+	9.03	92
37	Employment to population ratio, 15+, female (%) (national estimate)	+	+	9.04	85
15	Income share held by lowest 10%	+	+	9.08	92
14	Income share held by highest 20%	+	+	9.2	92
13	Income share held by highest 10%	+	+	9.21	92
24	Poverty headcount ratio at national poverty lines (% of population)	+	+	9.46	75
39	Employment to population ratio, ages 15-24, female (%) (national estimate)	+	+	9.57	80
63	Number of households 5 persons - Proportion over All households	+	+	11.15	56
53	Number of households All households - Per capita	+	+	11.51	61
57	Number of households 2 persons - Proportion over All households	+	+	11.59	56
62	Number of households 5 persons - Per capita	+	+	11.64	56
56	Number of households 2 persons - Per capita	+	+	11.66	56
54	Number of households 1 person - Per capita	+	+	11.82	56
55	Number of households 1 person - Proportion over All households	+	+	11.89	56
58	Number of households 3 persons - Per capita	+	+	11.94	56
65	Number of households 6 persons and over - Proportion over All households	+	+	11.96	53
64	Number of households 6 persons and over - Per capita	+	+	12.07	53
59	Number of households 3 persons - Proportion over All households	+	+	12.09	56
60	Number of households 4 persons - Per capita	+	+	12.19	56
61	Number of households 4 persons - Proportion over All households	+	+	12.26	56
66	Total population in a household, both sexes	-	+	17.4	26
49	Number of doctors' consultations	+	+	26.21	25
51	Average number of people per room in occupied housing unit	-	+	33.32	12

Table B: Presence or absence of the two studies with highest value of R_0 from non-imputed random forest 4-fold cross validation experiments

	Chile (rural), 1967-68	Czech Republic, <1967	Average MSE over 10 repetitions	Number of studies with valid values
Using all indicators with no missing values	+	+	9.87	98
Using all indicators with up to 10 missing values	+	-	8.19	69
Using all indicators with up to 20 missing values	+	-	9.6	52
Using all indicators with up to 40 missing values	+	-	13.8	32
Using all indicators with up to 70 missing values	-	-	6.88	20

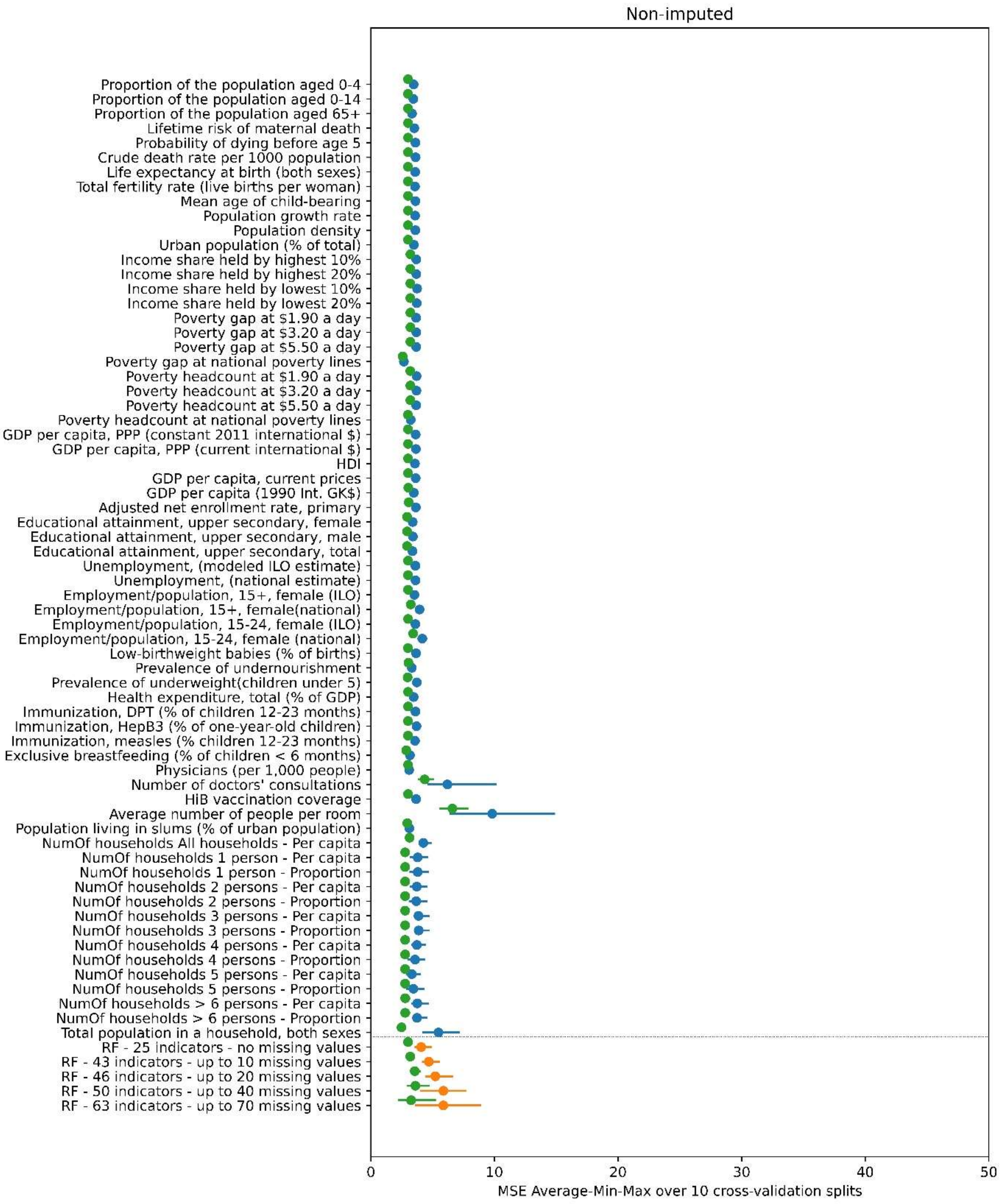

Fig A: Similar to Fig 3 of the main text but with the two studies ('Czech Republic, <1967' and 'Chile (rural), 1967-68') excluded. Mean value (blue and orange dots) and minimum-maximum value range (blue and orange line) of the MSE of the predicted R_0 over ten 4-fold cross validation splits

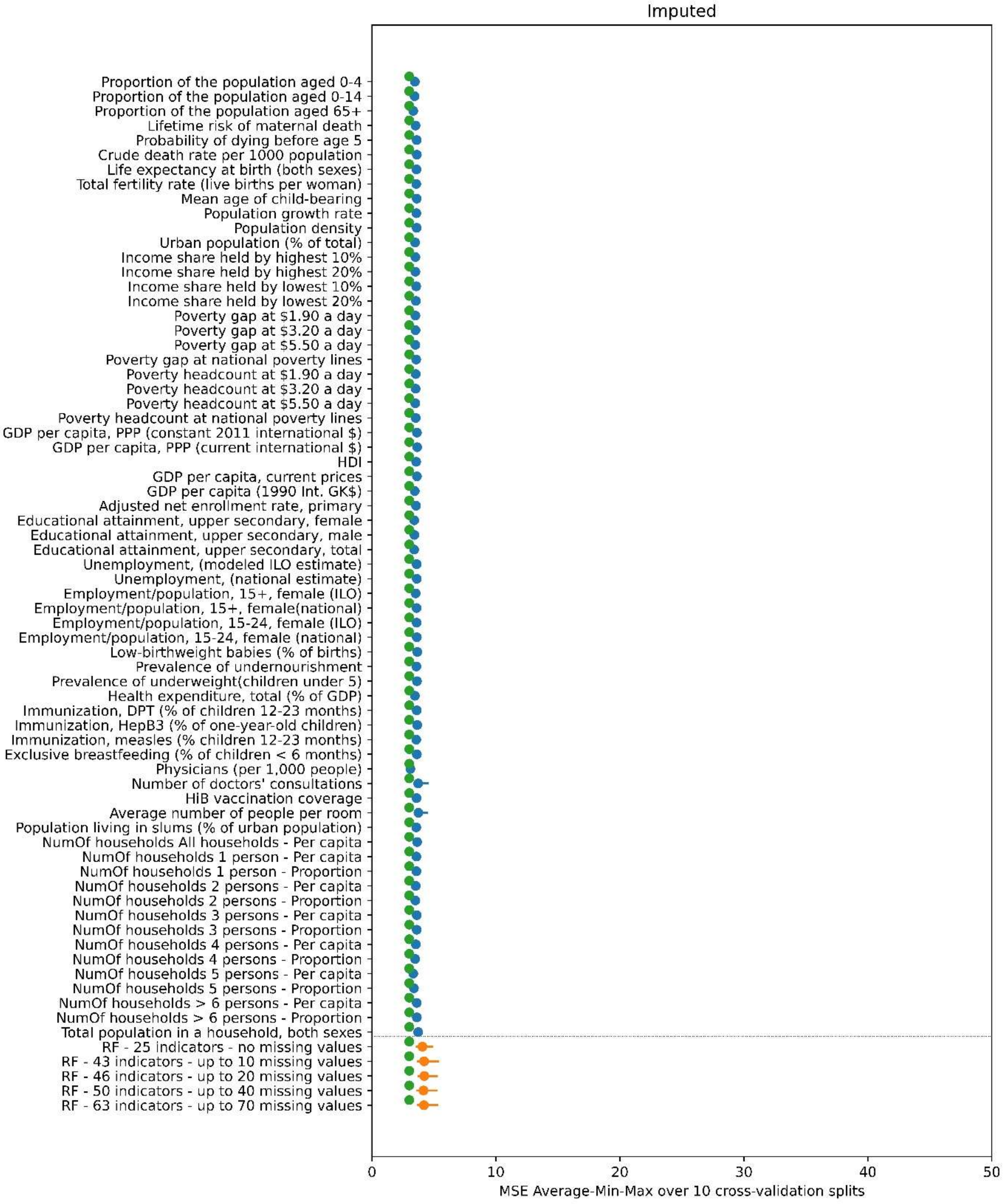

Fig B: Similar to Fig 4 of the main text but with the two studies ('Czech Republic, <1967' and 'Chile (rural), 1967-68') excluded. Mean value (blue and orange dots) and minimum-maximum value range (blue and orange line) of the MSE of the predicted R_0 over ten 4-fold cross validation splits
