# Supplementary material for: Estimates of the basic reproduction number for rubella using seroprevalence data and indicator-based approaches
Source: PLoS Comput Biol. 2022 Mar 3;18(3):e1008858. doi: 10.1371/journal.pcbi.1008858 (PMC8893344; doi:10.1371/journal.pcbi.1008858)
Supplement: S3 Text — Table A: MSE performance and rank of the 96 parameter sets in the double-loop nested cross-validation experiment. The default parameter setup is in bold font. (PDF) [file pcbi.1008858.s008.pdf]

Random forest parameter tuning

For the parameter optimisation results we present in the main test we run a nested cross-validation experiment over a grid spanning 6 parameters and a total of 96 points. We chose these parameters to include following the description in the documentation of scikit-learn documentation (https://scikit-learn.org/stable/modules/ensemble.html). The 6 parameters together with their default value (in brackets) and their alternative values are listed below:

- ‘bootstrap’: [True], False
- ‘max_depth’: [None], 10
- ‘max_features’: [‘auto’], ‘sqrt’
- ‘min_samples_leaf’: [1], 2
- ‘min_samples_split’: [2], 4
- ‘n_estimators’: [100], 25, 200

In Table A we list the MSE (average, minimum and maximum over the 10 splits) obtained from the nested cross-validation optimisation run for the 96 points of the parameters grid. We also list the rank (average, minimum and maximum over the 10 splits) in terms of the achieved MSE for each grid point. For the ‘bootstrap’ and ‘max_features’ parameters for which the ‘TRUE and ‘sqrt’ values are performing consistently better. For the other 4 parameters there does not seem to be a clear separation between better and worse performing values.

Table A: MSE performance and rank of the 96 parameter sets in the double-loop nested cross-validation experiment. The default parameter setup is in bold font.

**bootstrap**	**max_depth**	**max_features**	**min_samples_leaf**	**min_samples_split**	**n_estimators**	**Mean MSE OverSplits**	**Min MSE OverSplits**	**Max MSE OverSplits**	**Mean Rank OverSplits**	**MinRankOverSplits**	**MaxRankOverSplits**
TRUE	10	sqrt	2	2	200	8.41	8.13	8.70	4.5	1	11
TRUE	10	sqrt	2	4	200	8.41	8.13	8.70	4.5	1	11
TRUE	None	sqrt	2	2	200	8.41	8.13	8.73	4.6	1	9
TRUE	None	sqrt	2	4	200	8.41	8.13	8.73	4.6	1	9
TRUE	None	sqrt	2	2	100	8.46	8.10	8.76	6	1	11
TRUE	None	sqrt	2	4	100	8.46	8.10	8.76	6	1	11
TRUE	10	sqrt	2	2	100	8.46	8.10	8.75	7.5	3	12
TRUE	10	sqrt	2	4	100	8.46	8.10	8.75	7.5	3	12
TRUE	None	sqrt	2	2	25	8.58	8.05	9.01	11.3	1	33
TRUE	None	sqrt	2	4	25	8.58	8.05	9.01	11.3	1	33
TRUE	10	sqrt	2	2	25	8.60	8.05	8.92	12.1	1	30
TRUE	10	sqrt	2	4	25	8.60	8.05	8.92	12.1	1	30
TRUE	None	sqrt	1	4	200	8.78	8.43	9.14	16.1	9	26
TRUE	10	sqrt	1	4	200	8.78	8.46	9.16	17.2	9	28
TRUE	None	sqrt	1	4	100	8.81	8.53	9.36	17.3	13	25
TRUE	10	sqrt	1	4	100	8.80	8.53	9.37	17.9	10	28
TRUE	10	auto	2	2	200	8.99	8.41	9.64	22.5	13	33
TRUE	10	auto	2	4	200	8.99	8.41	9.64	22.5	13	33
TRUE	None	auto	2	2	200	8.99	8.41	9.64	22.9	15	31
TRUE	None	auto	2	4	200	8.99	8.41	9.64	22.9	15	31
TRUE	10	auto	2	2	100	9.04	8.52	9.84	23.7	11	37
TRUE	10	auto	2	4	100	9.04	8.52	9.84	23.7	11	37
TRUE	None	auto	2	2	100	9.04	8.54	9.85	24.4	13	37
TRUE	None	auto	2	4	100	9.04	8.54	9.85	24.4	13	37
TRUE	10	sqrt	1	4	25	9.03	8.47	9.61	25.7	9	48
TRUE	None	sqrt	1	4	25	9.02	8.49	9.63	26.3	14	50
TRUE	None	sqrt	1	2	200	9.09	8.62	9.67	27	17	36
TRUE	10	sqrt	1	2	200	9.11	8.60	9.70	27.6	14	37
TRUE	None	sqrt	1	2	100	9.12	8.62	9.85	28	16	37
TRUE	10	sqrt	1	2	100	9.15	8.64	9.94	28.4	10	38
TRUE	None	auto	2	2	25	9.28	8.69	10.51	34.3	3	59
TRUE	None	auto	2	4	25	9.28	8.69	10.51	34.3	3	59
TRUE	10	auto	2	2	25	9.28	8.65	10.49	34.6	1	55
TRUE	10	auto	2	4	25	9.28	8.65	10.49	34.6	1	55
TRUE	10	sqrt	1	2	25	9.35	8.47	10.23	36.3	14	47
TRUE	None	sqrt	1	2	25	9.36	8.29	10.27	37	13	58
TRUE	10	auto	1	4	100	9.54	8.63	10.61	41	18	54
TRUE	None	auto	1	4	100	9.54	8.66	10.60	41.4	23	53
TRUE	10	auto	1	4	25	9.64	8.55	11.55	41.8	15	68

TRUE	None	auto	1	4	200	9.55	8.74	10.37	41.8	15	55
TRUE	None	auto	1	4	25	9.63	8.60	11.53	41.9	22	67
TRUE	10	auto	1	4	200	9.55	8.77	10.39	42	16	56
FALSE	None	sqrt	2	2	100	9.57	9.11	10.27	43.9	27	54
FALSE	None	sqrt	2	4	100	9.57	9.11	10.27	43.9	27	54
FALSE	10	sqrt	2	2	100	9.57	9.11	10.16	44.1	25	55
FALSE	10	sqrt	2	4	100	9.57	9.11	10.16	44.1	25	55
FALSE	None	sqrt	2	2	200	9.62	9.10	10.33	45.4	31	59
FALSE	None	sqrt	2	4	200	9.62	9.10	10.33	45.4	31	59
FALSE	10	sqrt	2	2	200	9.61	9.11	10.19	45.5	29	60
FALSE	10	sqrt	2	4	200	9.61	9.11	10.19	45.5	29	60
FALSE	10	sqrt	2	2	25	9.63	9.22	10.13	47.3	21	62
FALSE	10	sqrt	2	4	25	9.63	9.22	10.13	47.3	21	62
FALSE	None	sqrt	2	2	25	9.66	9.02	10.10	47.8	27	65
FALSE	None	sqrt	2	4	25	9.66	9.02	10.10	47.8	27	65
TRUE	None	auto	1	2	200	9.85	8.87	10.73	50.5	27	60
TRUE	10	auto	1	2	200	9.85	8.92	10.76	50.7	32	60
TRUE	10	auto	1	2	100	9.85	9.00	11.01	51.1	36	60
TRUE	None	auto	1	2	100	9.87	8.95	11.11	51.4	35	61
TRUE	None	auto	1	2	25	10.00	8.87	12.13	52	38	72
TRUE	10	auto	1	2	25	9.98	8.91	11.79	52.2	40	71
FALSE	None	sqrt	1	4	100	10.41	9.18	11.67	59.5	49	64
FALSE	None	sqrt	1	4	200	10.42	9.21	11.58	60.1	51	64
FALSE	10	sqrt	1	4	200	10.44	9.25	11.46	60.6	52	66
FALSE	10	sqrt	1	4	100	10.46	9.27	11.53	61.1	53	67
FALSE	None	sqrt	1	4	25	10.65	9.40	11.75	62.9	59	66
FALSE	10	sqrt	1	4	25	10.78	9.46	12.21	64.3	61	66
FALSE	None	sqrt	1	2	100	11.18	9.84	12.58	69.3	64	82
FALSE	None	sqrt	1	2	200	11.22	9.79	12.62	69.5	63	83
FALSE	10	sqrt	1	2	200	11.22	9.76	12.66	69.6	65	81
FALSE	10	sqrt	1	2	100	11.22	9.81	12.87	69.8	66	80
FALSE	None	sqrt	1	2	25	11.46	9.99	13.04	71.8	67	84
FALSE	10	sqrt	1	2	25	11.46	9.84	13.27	72.7	65	84
FALSE	10	auto	2	2	25	13.16	11.36	18.22	80.8	71	91
FALSE	10	auto	2	4	25	13.16	11.36	18.22	80.8	71	91
FALSE	None	auto	2	2	25	13.14	11.42	18.00	80.8	71	93
FALSE	None	auto	2	2	100	13.18	11.38	18.27	80.8	73	89
FALSE	None	auto	2	4	25	13.14	11.42	18.00	80.8	71	93
FALSE	None	auto	2	4	100	13.18	11.38	18.27	80.8	73	89
FALSE	10	auto	2	2	100	13.18	11.29	18.32	81.4	73	95
FALSE	10	auto	2	4	100	13.18	11.29	18.32	81.4	73	95
FALSE	10	auto	2	2	200	13.19	11.24	18.41	81.6	67	95
FALSE	10	auto	2	4	200	13.19	11.24	18.41	81.6	67	95
FALSE	None	auto	2	2	200	13.19	11.30	18.42	81.6	69	95

FALSE	None	auto	2	4	200	13.19	11.30	18.42	81.6	69	95
FALSE	10	auto	1	4	25	14.99	10.81	19.30	81.8	73	90
FALSE	None	auto	1	4	25	14.97	10.65	19.06	82.7	73	89
FALSE	None	auto	1	4	200	15.02	10.61	19.07	83.6	73	89
FALSE	10	auto	1	4	200	15.05	10.71	19.24	83.8	76	90
FALSE	10	auto	1	4	100	15.07	10.69	19.27	84	76	90
FALSE	None	auto	1	4	100	15.03	10.62	19.05	84.1	74	90
FALSE	10	auto	1	2	25	16.23	11.16	21.21	89.1	80	96
FALSE	None	auto	1	2	25	16.26	11.22	21.05	89.3	79	96
FALSE	None	auto	1	2	200	16.27	11.10	21.17	89.8	79	95
FALSE	None	auto	1	2	100	16.29	11.10	21.18	90.1	79	96
FALSE	10	auto	1	2	200	16.31	11.22	21.38	90.2	81	96
FALSE	10	auto	1	2	100	16.33	11.25	21.43	90.9	83	96
