# Supplementary material for: Estimates of the basic reproduction number for rubella using seroprevalence data and indicator-based approaches
Source: PLoS Comput Biol. 2022 Mar 3;18(3):e1008858. doi: 10.1371/journal.pcbi.1008858 (PMC8893344; doi:10.1371/journal.pcbi.1008858)
Supplement: S4 Text — Including or excluding the force of infection bootstraps of a study in the calculation of its default R0 estimate Fig A: Estimates of the basic reproduction number for each of the studies in the EMRO region. The blue and red circles and bars are identical as in Fig 1 of the main text. The black circles and ranges are the default R0 estimates and 95% CI that we obtain for each study when we exclude bootstrap values from that study from the collection of 1000 force of infection bootstrap used in the calculation. Table A: Comparison of default R0 values with the values obtained when the regional collection of 1000 force of infections bootstrap values does not include those coming from the study for which the default R0 is calculated. (PDF) [file pcbi.1008858.s009.pdf]

Including or excluding the force of infection bootstraps of a study in the calculation of its default R_0 estimate

As is discussed in more detail in the Methods and the Discussion sections of the main text, following the practice used in previous studies [1-4] , our calculation of the default R_0 estimate of each study is based on the collection of 1000 force of infection estimates from all the studies in the same geographical region as the study, with this collection including some force of infections bootstrap values from that same study. In order to comparatively examine the impact of that, we also calculated what the default R_0 estimate would be for each of the studies in one region (EMRO region) if the collection of 1000 force of infection bootstrap estimates used for each study only included bootstrap values from the other studies. The results of this calculation are plotted in Fig A and Table A below.

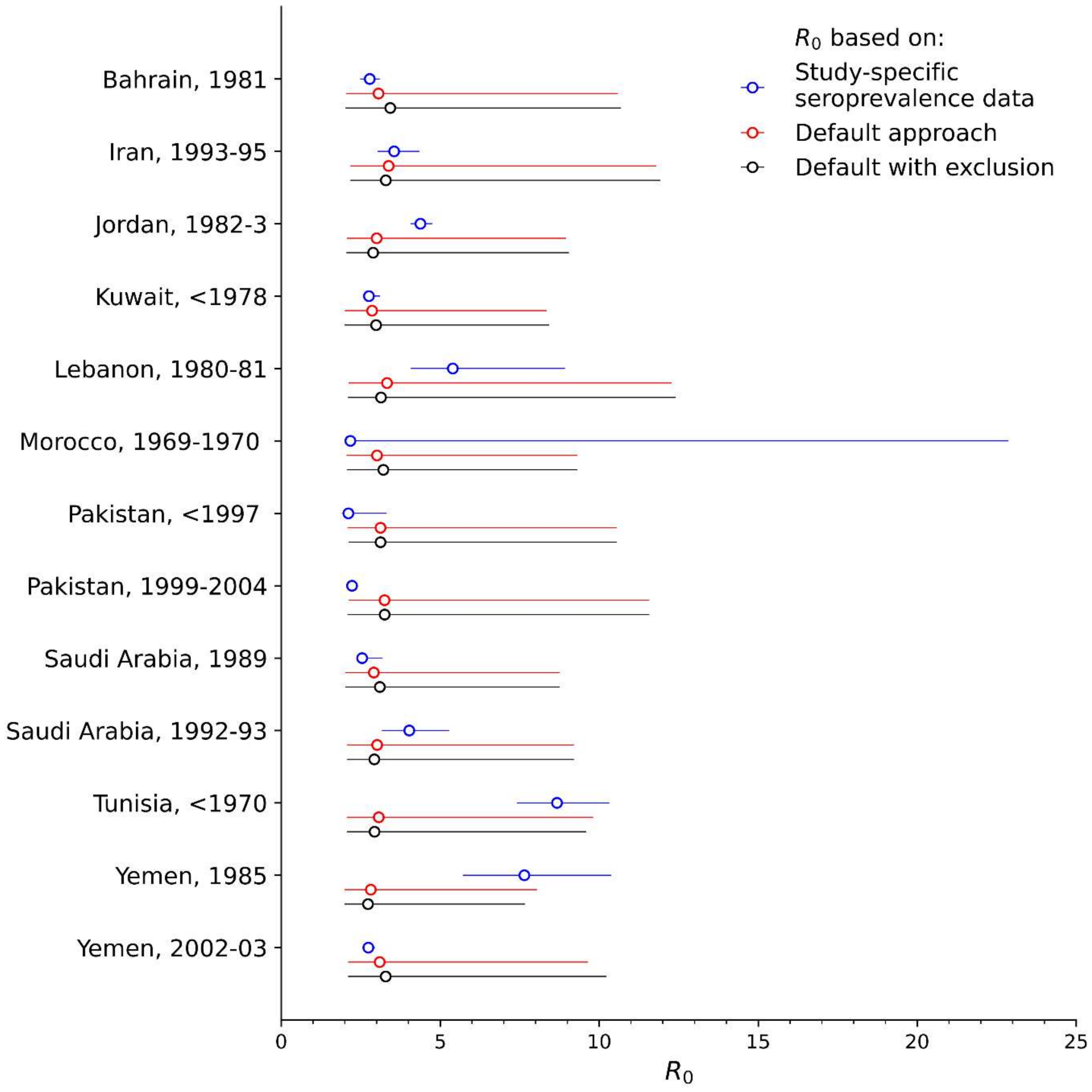

Fig A: Estimates of the basic reproduction number for each of the studies in the EMRO region. The blue and red circles and bars are identical as in Fig 1 of the main text. The black circles and ranges are the default R_0 estimates and 95% CI that we obtain for each study when we exclude bootstrap values from that study from the collection of 1000 force of infection bootstrap used in the calculation.

Table A: Comparison of default R_0 values with the values obtained when the regional collection of 1000 force of infections bootstrap values does not include those coming from the study for which the default R_0 is calculated.

	Default approach			Default with exclusion			
Study name (as in EMRO region column of Table A in S1 Table)	2.5%	50% (default R_0)	97.5%	2.5%	50% (default R_0)	97.5%	Relative difference of default R_0
Bahrain, 1981	2.035	3.056	10.588	2.022	3.425	10.689	12.07%
Iran, 1993-95	2.177	3.374	11.787	2.174	3.287	11.920	2.57%
Jordan, 1982-3	2.060	2.997	8.959	2.050	2.889	9.047	3.62%
Kuwait, <1978	2.000	2.855	8.343	1.990	2.981	8.420	4.39%
Lebanon, 1980-81	2.112	3.324	12.278	2.097	3.136	12.404	5.67%
Morocco, 1969-1970	2.054	3.009	9.307	2.057	3.208	9.307	6.60%
Pakistan, <1997	2.080	3.121	10.553	2.122	3.121	10.553	0.00%
Pakistan, 1999-2004	2.122	3.246	11.577	2.081	3.250	11.577	0.13%
Saudi Arabia, 1989	2.015	2.910	8.758	2.015	3.097	8.758	6.41%
Saudi Arabia, 1992-93	2.061	3.014	9.206	2.061	2.923	9.206	3.00%
Tunisia, <1970	2.065	3.063	9.811	2.063	2.934	9.593	4.24%
Yemen, 1985	1.985	2.814	8.051	1.985	2.726	7.658	3.14%
Yemen, 2002-03	2.101	3.095	9.653	2.101	3.282	10.232	6.02%

References

1. Vynnycky E, Papadopoulos T, Angelis K. The impact of Measles-Rubella vaccination on the morbidity and mortality from Congenital Rubella Syndrome in 92 countries. Hum Vaccin Immunother. 2019;15(2):309-16. Epub 2018/10/05. doi: 10.1080/21645515.2018.1532257. PubMed PMID: 30285537; PubMed Central PMCID: PMCPMC6422479.
2. Lee LA, Franzel L, Atwell J, Datta SD, Friberg IK, Goldie SJ, et al. The estimated mortality impact of vaccinations forecast to be administered during 2011-2020 in 73 countries supported by the GAVI Alliance. Vaccine. 2013;31 Suppl 2:B61-72. Epub 2013/04/26. doi: S0264-410X(12)01628-3 [pii] 10.1016/j.vaccine.2012.11.035. PubMed PMID: 23598494.
3. Toor J, Echeverria-Londono S, Li X, Abbas K, Carter ED, Clapham HE, et al. Lives saved with vaccination for 10 pathogens across 112 countries in a pre-COVID-19 world. Elife. 2021;10. Epub 2021/07/14. doi: 10.7554/eLife.67635. PubMed PMID: 34253291; PubMed Central PMCID: PMCPMC8277373.
4. Li X, Mukandavire C, Cucunuba ZM, Echeverria Londono S, Abbas K, Clapham HE, et al. Estimating the health impact of vaccination against ten pathogens in 98 low-income and middle-income countries from 2000 to 2030: a modelling study. Lancet. 2021;397(10272):398-408. Epub 2021/02/01. doi: 10.1016/S0140-6736(20)32657-X. PubMed PMID: 33516338; PubMed Central PMCID: PMCPMC7846814.
