# Supplementary material for: Estimates of the basic reproduction number for rubella using seroprevalence data and indicator-based approaches
Source: PLoS Comput Biol. 2022 Mar 3;18(3):e1008858. doi: 10.1371/journal.pcbi.1008858 (PMC8893344; doi:10.1371/journal.pcbi.1008858)
Supplement: Attachment — Submitted filename: ResponsesAndChanges.docx [file pcbi.1008858.s010.docx]

**Response to reviewer comments**

We thank the reviewers for their helpful comments. We have included the reviewers’ comments below (in italic font) and our responses are in normal font. Also, to improve readability, we have included some of the revised parts of the manuscript (in blue font). Sentences that are inserted in the old version are highlighted in yellow.

**Reviewer 1**: “*The authors attempt to understand the variation in rubella’s basic reproduction number in countries for which pre-vaccination serological surveys are available in terms of measurable population characteristics. This is a thoughtful, carefully conducted, and important study with however disappointing results. Urban and rural differences would be expected by virtue of the relationship between contact rates and population density, and other patterns (e.g., education, healthcare, ...) probably are related. Other social phenomena should have greater impact than observed.*

*Some thoughts:*

*We know that the basic reproduction number is a property of a pathogen in a host population. One possible explanation for the as-yet-unexplained variation is that the pathogen is genetically heterogeneous (Rivailler et al. J Gen Virol 2017; 98:396-404). Another is that host age is far too influential to distinguish only children and adults, a possibility that the synthetic matrices of Prem et al. (PLoS Comput Biol 2017; 13:e1005697) make testable, assuming that mixing patterns haven’t changed. A third possibility is that the different assays (Dimech et al. Clin Microbiol Rev 2016; 29:163-74) that almost certainly were used obscured meaningful patterns.*”

**Response to Reviewer 1**

We thank the reviewer for pointing out the merits of our work and also for offering these possible explanations for some of our results. We have included all these points in the revised version of the Discussion (page 19 of the revised manuscript) as copied below:

“The method we used to calculate the study-specific R0 estimates had a few limitations: Our analyses did not take into account the genetic diversity of the Rubella virus[9] which may be a source of variability of the basic reproduction number over different settings, that could not have been predicted by the indicator that we used as inputs to the machine learning methods we examined. Another limitation is that, while the seroprevalence data used to calculate the force of infection estimates used in the R0 typically spanned three or more age groups, we used two age groups to describe the age-mixing patterns. The influence of more complex mixing patterns has been described elsewhere [10]. Finally, the possible effect of different assays in the seroprevalence data that we used for the determination of the study-specific R0 estimates[11] may have introduced a source of variability not predictable by our machine learning methods.”

**Reviewer 2 Minor #1**: “*Results first paragraph, description of Figure 1 findings. Please clarify where you are describing the country-specific R0 estimates, potentially specifying these as median estimates from the bootstrapped FoI estimates. It was just a little confusing to refer to the country-specific as point-estimates since the figure uses the term “point-estimate” for the regional estimates.*”

**Response to Reviewer 2 Minor #1**

Many thanks for pointing this out. We apologise for the confusion. We have now made the definitions clearer and we have clarified the terminology. We have revised the section “Methods - Estimating the basic reproduction number” to better explain the different estimates of *R_0_* that we consider. We have introduced and defined the term “study-specific *R_0_*” which denotes the *R_0_* estimate based on a single estimate of the force of infection coming from a single seroprevalence study. This term now replaces the use of the expression “country-specific” in the text. The revised version of that section also now clarifies the definitions of the two estimates of *R_0_* obtained from regional collections of bootstrap-derived force of infection estimates (“default *R_0_*” and “regional point estimate of R_0_”). The corresponding parts of the revised version of “Methods - Estimating the basic reproduction number” (excerpts from pages 23-25) are copied below and the text that been added is highlighted in yellow:

For each of the 98 seroprevalence studies, point estimates of the force of infection among those aged <13 and ≥13 years, as published elsewhere[4, 5], were used to calculate the basic reproduction number, as the dominant eigenvalue of the Next Generation Matrix[1]. We refer to the estimates of *R_0_* derived from the point estimates of the force of infection from each study as “study-specific point estimates of *R_0_*”. Additionally, 1000 bootstrap-derived samples for the force of infection (calculated as described in[4]) were used to derive 1000 bootstrap-derived estimates of the study-specific *R_0_* and, subsequently, the 95% range of the study-specific *R_0_* for each study.

Previous studies have approximated the epidemiology of rubella in settings lacking seroprevalence by using a regionally averaging approach, using 1000 bootstrap-derived samples of the forces of infection compiled from all the studies from the same World Health Organization region[4, 5]. To evaluate this approach, for each of the 98 studies we calculated what the median *R_0_* and its 95% range would have been estimated to be from these 1000 bootstrap-derived force of infection samples drawn from all the countries of the corresponding region. The bootstraps drawn from each study to create the each of those regional collection of 1000 force of infection bootstrap values is described in detail in references[4, 5]. We refer to this median *R_0_* as the “default *R_0_*” throughout the text. These default estimates of *R_0_* for the 98 studies are the same throughout our analyses including the cross-validation results we present. The error associated with the default *R_0_* and that calculated using the study-specific point estimate of *R_0_* was quantified as the mean square error (MSE) over the 98 studies. The 95% range of the MSE was calculated using 1000 bootstrap-derived estimates of the MSE computed from the corresponding *R_0_* estimates that were computed from 1000 bootstrap-derived force of infection.

In sensitivity analyses, we also calculate what *R_0_* for a given setting would have been estimated to be using a point estimate of the age-dependent force for the region, which has previously been used to obtain an approximate estimate of the average CRS incidence for countries lacking seroprevalence data[5, 8]. This regional point estimate of the force of infection was taken as the value which led to the median burden of CRS out of the 1000 bootstraps in the absence of vaccination (see supplement in ref[5]). We refer to the estimate of the basic reproduction number obtained using the regional point estimate of the force of infection as the “regional point estimate of *R_0_*”

**Reviewer 2 Minor #2**: “*Results second paragraph. I think the first sentence needs to be revised to say that R0 was higher (rather than lower) for urban than for rural regions.*”

**Response to Reviewer 2 Minor #2**

We thank the reviewer for bringing this error to our attention. We have now fixed it.

**Reviewer 2 Minor #3**: “*Results third paragraph, first sentence - Region of the Americas is listed twice.*”

**Response to Reviewer 2 Minor #3**

We thank the reviewer for bringing this error to our attention. We have now fixed it.

**Reviewer 2 Minor #4**: “*Page 10, second paragraph within section on correlation b/w R0 calculated using country-specific seroprevalence data and indicators - the sentence reads, “When the simple linear regression was repeated by region, economic indicators were the most correlated with the basic reproduction number in Africa (Table 2).” Is it rather... when the simple correlation analyses were repeated by region ...?*”

**Response to Reviewer 2 Minor #4**

We thank the reviewer for bringing this error to our attention. We have now fixed it.

**Reviewer 2 Major #1 (part 1)**: “*The broad findings from the work are based on the comparison of the prediction methods to the regional averaging (default) approach. I think the paper would benefit from a somewhat clearer understanding of the default approach. Specifically, what bootstrap-derived estimates of the force of infection are relied on in each analysis? In the non-imputed analysis* *described in S3 are the bootstrapped derived FoI estimates only from the settings in the three training folds, and does that explain why the MSE estimates vary across indicator in the non-imputed analysis compared to the imputed analysis?*”

**Response to Reviewer 2 Major #1 (part 1)**

We apologise for the lack of clarity. In the default approach, the same set of bootstrap-derived FOI estimates is used to calculate R_0_, irrespective of the training fold and whether or not we were performing imputed or non-imputed analyses. The R_0_ values used and their 95% range are represented by the red circles and bars respectively in Figure 1. As such, the method used to calculate the default R0 does not explain the variation in the MSE. The variation in the MSE is explained, however, by the analysis we include in section “S6 Results – Effect of studies with high R0 values on the performance of simple linear regression and Random Forest prediction” of the supplementary material. We have clarified the method used to calculate R_0_ using the default approach in the section “Methods - Estimating the basic reproduction number”, as described in the response to reviewer 1 (see above).

**Reviewer 2 Major #1 (part 2)**: “*If true, are the FoI (from which to estimate a median R0) resampled 1000 times from the settings in the training folds, or are the FoI only a subset of the original 1000 bootstrapped samples? Also if true, how does this align with the discussion that states "The performance of this approach may have been overestimated given that bootstrap-derived estimates used to calculate the median would have included those from each of the studies being used to evaluate the performance," which reads as if bootstrap-derived estimates of the force of infection sampled from all 98 study sites were included in all default MSE estimates?*”

**Response to Reviewer 2 Major #1 (part 2)**

These questions are addressed in the preceding response, since we did not use the approach suggested by the reviewer.

**Reviewer 2 Major #2**: “*On a similar note, if indeed bootstrap-derived estimates of the force of infection sampled from all 98 study sites were included in all default estimates, this does seem to be a relatively systematic bias that would decrease the MSE. Is it possible to conduct a sensitivity analysis for at least one indicator or subset of indicators assessing R0 MSE if you didn't include FoI estimates from the study site you are trying to predict?*”

**Response to Reviewer 2 Major #2**

We acknowledge the reviewer’s point that, by the way it is calculated, the default estimate may have an advantage in its cross-validation comparison with the machine learning methods. We have included a paragraph in the Discussion section (page 19 of the revised manuscript) which acknowledges this point.

We have also included a small scale analysis (see results for EMRO region in section S8 of the Supplementary material and related part in the revised version of the Discussion section) where we compare the values of the default *R_0_* estimate with and without excluding the force of infection bootstraps coming from the particular study for which we are estimating the default *R_0_*. The difference in the *R_0_* values estimated from the two approaches is very small, with the values differing by less than 5% in 8 of the 13 cases considered, by up to 6.6% in 4 more cases and by 12% in one case.

We also wish to point out that to design an analysis that is comparable with the other cross-validation results in the paper we would have to make different default *R_0_* calculations which, for each study and each split to folds, exclude from the force of infection bootstrap collection not only the particular study but all the studies that are in the test set of that particular split to folds. Taking also into account the complications that arise from the missing indicator values in the linear regression and the need to exclude certain splits to folds that do not have enough studies with valid indicator values in the test set, we would now have to make sure that for all studies in all the test folds, the corresponding training sets contain at least one study from the corresponding region. This was beyond the scope of our current analyses and for simplicity, we have not included it in this paper but have expanded the appropriate paragraph in the discussion section to discuss this limitation. We copy this paragraph below:

In a related manner, the comparison of the performance of the default approach to those of the Machine Learning methods using a cross-validation methodology is possibly giving an advantage to the former. This is because the default *R_0_* estimates for each study are calculated once, prior to the cross-validation split to folds, and they make use of information in studies from the same region regardless of whether these fall in the test or training set in each particular split to folds. A more accurate version of that comparison would be to modify the default approach so that for each different indicator and each different split to cross-validation folds, a different default *R_0_* estimate is calculated for each study by excluding from the regional collection of force of infection bootstraps the studies that are in the same test fold (this exclusion would be dependent on the different indicators because of the missing values issue). However, given the minimal impact of excluding force of infection estimates of one study at a time (see discussion above and section S8), and the increased complexity of the aforementioned modification, we did not pursue it.

**Reviewer 2 Major #3**: “*The authors state in the results that “For both assumptions about contact, estimates of R0 using the regional point estimate of the force of infection were usually smaller and led to a larger mean square error than the default R0 (Fig 1, Fig S2 and Table S5, Supplement),” but the significance of this finding is never revisited. It is possible to include a brief description of the motivation for this analysis and potentially the broad take away of this estimate under-performing compared to the regional averaging approach?*”

**Response to Reviewer 2 Major #3**

We thank the reviewer for pointing this out. We are copying below the excerpt from the revised version of the Discussion section of the manuscript (page 17) in which we are addressing this point:

To our knowledge, our analyses are the first to assess the performances of regional averaging (default), simple linear regression and random forest approaches in predicting *R_0_*. We found that the default regional averaging approach employed in this work, whereby we calculated a set of *R_0_* estimates based on each force of infection estimate from the regional collection of bootstraps and then took the median of that *R_0_* estimate, performed better than the “regional point estimate of *R_0_*”. The under-performance of the latter is unsurprising, given its method of computation using the regional “point estimates” of the force of infection. These were defined in previous analyses[5, 8-10], in which the main outcome of interest of interest was the incidence of Congenital Rubella Syndrome (CRS), as the force of infection estimates which led to the median incidence of CRS per 100,000 live births in the absence of vaccination. Since the CRS incidence depends both on the proportion of women who reach child-bearing age still susceptible and the force of infection among adult women, the combination of force of infection estimates among children and adults, out of 1000 bootstrap-derived values, which results in the median CRS incidence is unlikely to result in the median estimate of the basic reproduction number.

**Reviewer #2: Have the authors made all data and (if applicable) computational code underlying the findings in their manuscript fully available?:** “*No: The authors include references to data (that would need to be extrapolated), pseudocode, and in depth details of their methods, but do not actually include the data and code that can be used to easily replicate their findings.*”

**Response to Reviewer 2**

We have uploaded all the related data and Jupyter Python Notebooks to a GitHub repository which can be accessed at <https://github.com/timosp/Estimates-basic-reproduction-number-rubella.git>. We have included the link to that repository in the “Methods – Software” section of the revised manuscript
